# Comprehensive Effects of *Flowering Locus* T-Mediated Stem Growth in Tobacco

**DOI:** 10.3389/fpls.2022.922919

**Published:** 2022-06-16

**Authors:** Jun Wu, Qiuhong Wu, Zhongjian Bo, Xuli Zhu, Junhui Zhang, Qingying Li, Wenqing Kong

**Affiliations:** ^1^Key Laboratory of Bio-Resources and Eco-Environment of Ministry of Education, College of Life Sciences, Sichuan University, Chengdu, China; ^2^Microbiology and Metabolic Engineering Key Laboratory of Sichuan Province, Chengdu, China; ^3^College of Biological Sciences and Biotechnology, Beijing Forestry University, Beijing, China

**Keywords:** *Flowering locus T*, *Jatropha curcas*, network regulation, transcriptome analysis, tobacco stem

## Abstract

In flowering plants, *Flowering locus T* (*FT*) encodes a major florigen. It is a key flowering hormone in controlling flowering time and has a wide range of effects on plant development. Although the mechanism by which *FT* promotes flowering is currently clearly understood, comprehensive effects of the *FT* gene on plant growth have not been evaluated. Therefore, the effects of FT on vegetative growth need to be explored for a complete understanding of the molecular functions of the *FT* gene. In this study, the *Jatropha curcas* L. *FT* gene was overexpressed in tobacco (JcFT^OE^) in order to discover multiple aspects and related mechanisms of how the *FT* gene affects plant development. In JcFT^OE^ plants, root, stem, and leaf development was strongly affected. Stem tissues were selected for further transcriptome analysis. In JcFT^OE^ plants, stem growth was affected because of changes in the nucleus, cytoplasm, and cell wall. In the nucleus of JcFT^OE^ plants, the primary effect was to weaken all aspects of DNA replication, which ultimately affected the cell cycle and cell division. The number of stem cells decreased significantly in JcFT^OE^ plants, which decreased the thickness and height of tobacco stems. In the cell wall of JcFT^OE^ plants, hemicellulose and cellulose contents increased, with the increase in hemicellulose associated with up-regulation of xylan synthase-related genes expression. In the cytoplasm of JcFT^OE^ plants, the primary effects were on biogenesis of ribonucleoprotein complexes, photosynthesis, carbohydrate biosynthesis, and the cytoskeleton. In addition, in the cytoplasm of JcFT^OE^ plants, there were changes in certain factors of the core oscillator, expression of many light-harvesting chlorophyll *a*/*b* binding proteins was down-regulated, and expression of *fructose 1,6-bisphosphatase* genes was up-regulated to increase starch content in tobacco stems. Changes in the xylem and phloem of JcFT^OE^ plants were also identified, and in particular, xylem development was affected by significant increases in expression of *irregular xylem* genes.

## Introduction

*Flowering locus T* (*FT*) encodes a protein that has a central role in initial stages of angiosperm flowering. The protein regulates a complex hierarchical signal network and promotes differentiation of apical meristems into flowers (Turck et al., 2008; Pin and Nilsson, 2012). The FT-like proteins are globular proteins of the phosphatidylethanolamine binding protein family and are typically expressed in the phloem, are graft-transmissible, and can move to stem tips and effectively stimulate flowering (Putterill and Varkonyi-Gasic, 2016). In a photoperiod-dependent pathway, gigantea and CONSTANS (CO) proteins function together (Zeevaart, 2008) to induce transcription of *FT* genes in vascular bundles at leaf tips (Sawa and Kay, 2011). After translation, FT proteins are transported from veins in leaf blades to apical meristems through the phloem (Notaguchi et al., 2008). The FT proteins then combine with the bZIP transcription factor flowering locus D (FD) to form FD/FT heterodimer complexes (Wigge et al., 2005). Those complexes promote transformation from vegetative to reproductive growth *via* activation of the suppression of CO overexpression 1 (SOC1) and apetala 1 (AP1) proteins (Yoo et al., 2005; Corbesier et al., 2007). Moreover, two rice FT homolog Hd3a monomers bind C-terminal regions of dimeric 14-3-3 proteins to produce a complex that translocates to the nucleus and binds to the FD transcription factor. The florigen activation complex formed by FT, FD, and 14-3-3 proteins then induces transcription of downstream flowering-related genes, which leads to flowering (Taoka et al., 2011, 2013; Putterill and Varkonyi-Gasic, 2016).

In addition to flowering, FT-like proteins are also major regulators in developmental processes. Pin and Nilsson (2012) found that the *Populus FT2* gene directly or indirectly regulates transcriptional activity of genes that control cell division but could also have other roles. FT overexpression can induce stomatal opening *via* regulation of H^+^-ATPase activity in guard cells (Kinoshita et al., 2011). The *FT-like* genes in *Populus* may also be involved in regulating callose plug formation and therefore the ability of signals to move through pores and plasmodesmata to shoot apical meristems (Rinne et al., 2011). In addition to controlling flowering and fruit set, the precursor of the florigen in tomato also regulates termination of symptomatic meristems and leaf structures, suggesting the florigen precursor is not only a signal for flower development but is also a general systemic regulator of tomato growth (Shalit et al., 2009). Ectopic expression of the rice *Hd3a* gene in potato induces tubers under noninductive long days (Navarro et al., 2011).

Florigen is a generic growth-attenuating hormone in both leaf and stem meristems (Shalit-Kaneh et al., 2019). When transgenic Arabidopsis, maize, tomato, tobacco, and other model species overexpress the *FT* or *FT-like* genes, stem and leaves are smaller than those of the wild type. However, when expression of *FT-like* genes is suppressed, transgenic plants develop broader stems and significantly larger leaves than those of wild-type plants (Izawa et al., 2002; Meng et al., 2011; Li et al., 2015). Therefore, *FT* or *FT-like* genes can have a distinct effect on stem and leaf development, mainly in the inhibition of stem and leaf growth.

To date, *FT* homologs have been identified in many species, demonstrating general conservation of functions across gymnosperms and angiosperms (Notaguchi et al., 2008; Laurie et al., 2011). The *FT-like* genes have evolved many roles and have important effects on plant diversity, adaptability, and domestication (Blackman et al., 2010; Liu et al., 2016). Therefore, *FT-like* genes can drive plant evolution as a single essential gene, the evolution of which has played a central role in plant diversification and adaptation (Pin and Nilsson, 2012).

Although previous research on the regulation of flowering by *FT* has been very thorough (Jaeger and Wigge, 2007; Turck et al., 2008; Taoka et al., 2013; Ho and Weigel, 2014), most studies only identify one or a few aspects that are regulated by *FT* during plant growth (Kinoshita et al., 2011; Gao et al., 2016; Chen et al., 2021). A comprehensive analysis of the influence of *FT* on growth and development will increase understanding of the function and evolution of *FT* and its homologous genes, as well as utilization of those gene resources. In this study, an *FT* homolog from *Jatropha curcas* L. (*JcFT*) that encoded the Jatropha protein Heading Date 3A (Li et al., 2014) was overexpressed in tobacco. To identify the multiple effects of *FT* on plant growth and explore the regulatory networks, transcriptome analysis was combined with morphological observations and stem carbohydrate content determination. Some results from previous research were confirmed or further explained, but novel mechanisms of *FT* involvement in plant growth and development were also identified. We discovered *FT* overexpression affected gene expression in the cell wall, cytoplasm and nucleus, including some important biological processes such as DNA replication, cell cycle, hemicellulose and cellulose metabolism. Stem structure and composition were altered in *FT*-overexpressing plants. We also revealed the main reasons for the short and thin stems and the impaired cell wall and vascular development in *FT*-overexpressing plants.

## Materials and Methods

### Vector Construction, Plant Transformation, and Cultivation

**Figure 1:**
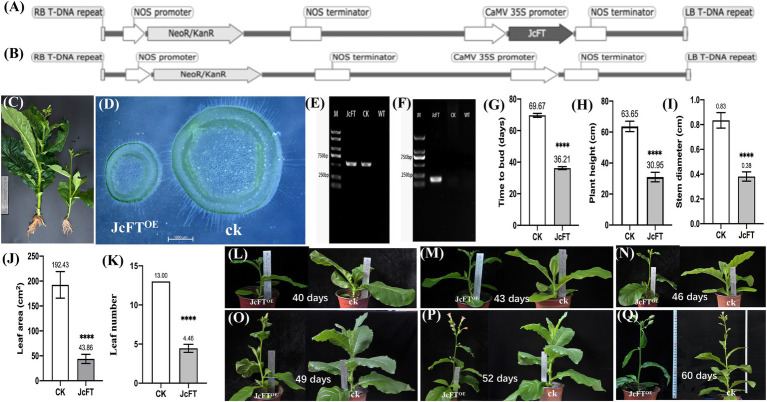
Traits of JcFT^OE^ transgenic and control plants. T-DNA of transformation vectors of **(A)** JcFT^OE^ and **(B)** the control. **(C)** Roots, plant height, leaf, stem thickness, and leaf number of JcFT^OE^ and control (CK) plants. **(D)** Stem cross sections of JcFT^OE^ and control plants. **(E,F)** Gel electrophoresis of PCR-amplified products used to identify transgenic tobacco (O, PCR amplification of *NPTII*; P, PCR amplification of *JcFT*). **(G–K)** Flowering time, plant height, stem diameter, leaf area and leaf number of JcFT^OE^ and control plants (the values represent mean, the vertical bars indicate standard deviation, *n* = 24, ^****^p < 0.0001). **(L–Q)** Different growth stages of JcFT^OE^ and control plants. Sampling from 43 to 49 days after seeding.

The gene *JcFT* (Accession: NM_001308752; Li et al., 2014) was overexpressed in *Nicotiana tabacum* “SR1.” The *JcFT* sequence was isolated from *J. curcas* and cloned into *SmaI* and *SacI* sites of the binary vector pBI121 (DNA Cloning Service, Genewiz, Suzhou, China). The constructed vector was designated as CaMV 35S::JcFT, and an empty vector was used as the control (Figures 1A,B). Constructs in binary vectors were introduced into *Agrobacterium tumefaciens* strain EHA105 and then transformed into wild-type *N. tabacum* “SR1” *via* a leaf disk method (Gallois and Marinho, 1995). Transgenic plants were identified *via* polymerase chain reaction (PCR) amplification of *aminoglycoside 3′*-*phosphotransferase* (*NPTII*) and *JcFT* genes using leaf DNA as the template. Primers for *NPTII* and *JcFT* amplification are shown in Supplementary Table 1. Plants were grown at 26°C under a 16-h light/8-h dark photoperiod. Transgenic seeds from the T5 generation were harvested and used in the study.

### RNA Isolation, Library Construction, and Sequencing

One hundred seeds of each *JcFT* transgenic tobacco and the control were sown simultaneously. Transgenic plants were confirmed using morphological characteristics and PCR analysis (Figures 1C–F). Thirty JcFT transgenic plants and 30 control plants were selected for further investigation. Although JcFT transgenic tobacco bloomed approximately 36 days after seeding, stems of JcFT transgenic tobacco and the control were too small, underdeveloped, and difficult to sample. Therefore, sampling began at 43 days after seeding. Middle sections of 18 stems (1–2 cm; remaining stem pieces were used in section analysis) were excised using an enzyme-free blade from healthy JcFT^OE^ and control plants. Three plants were sampled every 3 days, which were then mixed and ground as a single transcriptome sample. The samples were collected in triplicate.

Total RNA was isolated using TaKaRa MiniBEST Plant RNA Extraction reagents following the manufacturer’s instructions (Takara Bio, Inc., Dalian, China). Integrity and concentration of RNA were checked using a NanoDrop ND-1000 spectrophotometer (Thermo Scientific, Wilmington, DE, United States) and an Agilent 2100 Bioanalyzer (Agilent Technologies, Santa Clara, CA, United States). A NEBNext Poly (A) mRNA Magnetic Isolation Module (E7490, NEB, MA, United States) was used to isolate mRNA. The cDNA library was constructed using a NEBNext Ultra RNA Library Prep Kit for Illumina (E7530, NEB) and NEBNext Multiplex Oligos for Illumina (E7500, NEB) following the manufacturer’s instructions. In brief, the enriched mRNA was fragmented into approximately 200-nt RNA inserts, which were used to synthesize first and second-strand cDNA. End-repair/dA-tail and adaptor ligation were performed on double-stranded cDNA. Suitable fragments were isolated by Agencourt AMPure XP beads (Beckman Coulter, Inc., CA, United States) and then enriched by PCR amplification with the following conditions: initial denaturation at 98°C for 30 s; 14 cycles of denaturation at 98°C for 10 s, annealing at 65°C for 30 s, and extension at 72°C for 30 s; and final extension at 72°C for 5 min. Amplification ended with a 4°C hold. Last, the cDNA libraries of tobacco stems were sequenced on a flow cell using an Illumina HiSeq™ 2500 sequencing platform (Illumina, Inc., CA, United States).

### Global and Differential Gene Expression Analysis of RNA-Seq Data

To ensure accuracy of subsequent analyses, reads were first filtered to obtain clean reads by removing reads containing linkers and those of low quality (reads in which the proportion of undetermined bases exceeded 10% or the number of bases with a quality score ≤10 accounted for more than 50% of the entire read; Ewing et al., 1998). Clean reads were mapped to the tobacco cultivar TN90 genome^1^ using the HISAT2 program (Kim et al., 2015). StringTie software (Pertea et al., 2015) was used to construct transcripts and evaluate gene expression. Pearson correlation analysis was performed on expression levels of paired samples (Schulze et al., 2012), and the coefficient of correlation *r* was used to evaluate correlation strength. BLAST software (Altschul et al., 1997) and NR (non-redundant protein sequence database), Swiss-Prot, GO (gene ontology), COG (cluster of orthologous groups of proteins), KOG (clusters of orthologous groups for eukaryotic complete genomes), Pfam, and KEGG (kyoto encyclopedia of genes and genomes) databases were used to annotate the genes. DEGseq (Wang et al., 2010) was used to detect differentially expressed genes (DEGs) between sample groups according to the screening criteria of log2 fold change (FC) ≥ 2 and FDR (false discovery rate) < 0.01. Results of differential expression analysis and the interaction pairs included in the STRING database (Franceschini et al., 2013) were combined to construct a DEG interaction network. The constructed protein interaction network was imported into Cytoscape version 3.8.1 (Shannon et al., 2003) for visual analysis. The functional grouping network of terms or pathways of the DEG sets was further analyzed by ClueGO (only showing pathways with *p* ≤ 0.05; Bindea et al., 2009). Overrepresented GO terms in the network were identified and displayed as a network of significant GO terms using BiNGO (significance level of 0.05; Maere et al., 2005). Weighted gene co-expression network analysis (WGCNA; Maertens et al., 2018) was used to identify clusters (modules) of highly correlated DEGs.

### Reverse-Transcription Quantitative Polymerase Chain Reaction

Total RNA was extracted from fresh tissue using a plant RNA extraction kit (Chengdu biofit biotechnologies CO., LTD, Chengdu, China). First-strand cDNA was synthesized using a HiScript® III RT SuperMix for qPCR Kit with gDNA wiper (Vazyme, Nanjing, China) according to the manufacturer’s instructions. Reverse-transcription quantitative PCR (RT-qPCR) was performed using AceQ® qPCR SYBR green master mix (Vazyme) on a CFX Connect Real-Time System (Bio-Rad, CA, United States). Primers used in RT-qPCR are presented in Supplementary Table 1. The RT-qPCR was performed using three technical replicates and two independent biological replicates for each sample. Data were analyzed using the 2^–ΔΔCT^ method (Livak and Schmittgen, 2001). The *L25 ribosomal protein* (*L25*) and *Elongation factor 1a* (*EF-1a*) genes were used as internal reference genes (Schmidt and Delaney, 2010).

### Plant and Cell Morphology

Flowering time, plant height, stem diameter, and leaf area were determined for 12 transgenic and 12 control plants. Middle sections of stems were embedded in paraffin (Carlquist, 1982) and sectioned in either the transverse or longitudinal plane into 3-μm thick slices using a Leica RM2235 microtome (Nussloch, Germany). Sections were placed on glass slides, stained with plant safranin and fast green staining solution (Servicebio, Wuhan, China), and observed under a tissue panoramic imaging scanning system (Wisleap WS-10, Beijing, China). Observation of epidermal cells was based on the peel method (Zhu et al., 2016). In addition, sections were observed and photographed with an inverted optical microscope (Olympus IX71, Tokyo, Japan), and sizes of 400 pith cells of six JcFT^OE^ and six control plants were measured using Image-Pro Plus 6.0 (Mei et al., 2016). ANOVA was performed in IBM SPSS Statistics (version 23.0, NY, United States) with tests of normality and homogeneity of variance that met the prerequisites for ANOVA.

### Carbohydrate Content Analysis

Twenty-four stem samples (12 JcFT transgenic plants and 12 control plants), collected at the same time as the transcriptome samples, were air-dried and then dried to a constant weight at 80°C for analysis of carbohydrate content, including water-soluble sugars, water-soluble starch, cellulose, hemicellulose, lignin, and xylan. The anthrone-sulfuric acid colorimetric method was used to determine the content of water-soluble sugars and water-soluble starch following the Laurentin and Edwards (2003) method. The Van Soest method was used to determine the cellulose, hemicellulose, and lignin content in stems (Hindrichsen et al., 2006). Microsoft Excel was used for ANOVA.

## Results

### Overview of RNA-Seq Data

A total of 38.86 Gb of clean data were obtained from the transcriptome analysis of stems of three JcFT^OE^ and three control plants (NCBI Sequence Read Archive accession number: PRJNA811432). Clean data of each sample reached 6.29 Gb, and the Q30 base was greater than 91.76%. Of the clean reads, 94.79–95.19% mapped to the *N. tabacum* “TN90” genome, with 89.88–91.05% mapped to exons, 4.52–5.54% mapped to introns, and 4.30–4.92% mapped to intergenic regions. Sequencing output data of each sample are presented in Supplementary Table 2. Pearson coefficients of correlation (*r*) between the three JcFT^OE^ samples ranged from 0.8 to 0.95 and those of the three control samples from 0.89 to 0.92. The *r* values between JcFT^OE^ and control samples were lower (0.286–0.56). Therefore, the three JcFT^OE^ and three control samples were classified into one category (Supplementary Figure 1), suggesting that sampling and sequencing data produced consistent results.

### Effect of JcFT^OE^ on Tobacco Traits

Compared with control plants (*n* = 24), flower budding accelerated significantly in JcFT^OE^ tobacco plants, but plant height, leaf area, and stem thickness decreased significantly. Compared with controls, time to bud (36.21 days) and plant height (30.95 cm) decreased significantly in JcFT^OE^ plants (Figures 1G,H), whereas stem diameter (0.381 cm) decreased by more than twice (Figures 1D,I) and leaf area (43.864 cm^2^) decreased by more than four times (Figure 1J). In addition, numbers of leaves of JcFT^OE^ plants also decreased significantly (Figures 1C,K), and root development was relatively weak (Figure 1C). During the sampling period, the stems of JcFT^OE^ and control plants were increasing in thickness and height (Figures 1L–Q), which can well reflect the influence of the *JcFT* on stem growth.

### Biological Processes Affected in JcFT^OE^ Plants

**Figure 2:**
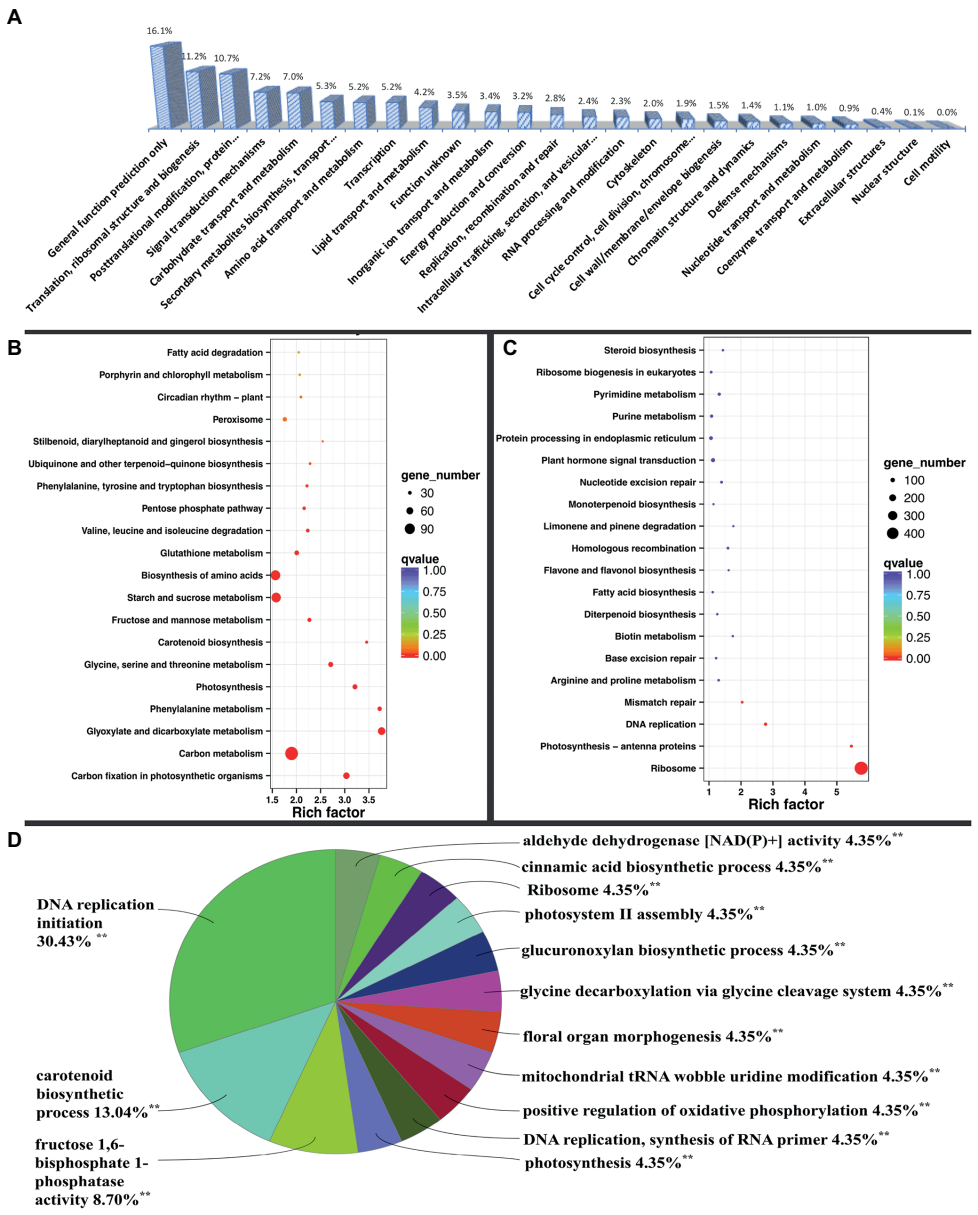
KOG (Eukaryotic Orthologous Groups classification) and ClueGO grouping of differentially expressed genes and KEGG (Kyoto Encyclopedia of Genes and Genomes) pathway enrichment analysis. **(A)** KOG classification of DEGs. KEGG pathway enrichment of **(B)** up-regulated and **(C)** down-regulated DEGs. **(D)** DEG ClueGO grouping of terms or pathways, with percentage referring to the proportion of annotated terms to the total terms in each group. ^**^Significantly annotated.

With overexpression of *JcFT*, there were 9,564 DEGs (FC ≥ 2 and FDR < 0.01) in JcFT^OE^ plants. The KOG (Eukaryotic Orthologous Groups) classification indicated that the DEGs were associated with 22 biological processes, including ribosomal structure and biogenesis, posttranslational modification, carbohydrate transport and metabolism, amino acid/lipid transport and metabolism, signal transduction, transcription, replication, inorganic ion transport, the cell cycle, cell division, and cell wall biogenesis, among others (Figure 2A). Of the DEGs, 5,516 were up-regulated and 4,048 were down-regulated. In the KEGG (Kyoto Encyclopedia of Genes and Genomes) pathway enrichment analysis, the up-regulated genes were significantly associated with carbon fixation, including carbon, glyoxylate, dicarboxylate, phenylalanine, starch, and sucrose metabolism; biosynthesis of carotenoids and amino acids; and photosynthesis (Figure 2B). The down-regulated DEGs were significantly associated with ribosomes, antenna proteins, and DNA replication (Figure 2C).

The DEGs were also analyzed using ClueGO in the Cytoscape software. Fourteen significant pathways or terms were annotated, including ribosome biogenesis, DNA replication initiation, carotenoid metabolic process, fructose 1,6-bisphosphate 1-phosphatase activity, photosynthesis, photosystem II assembly, and cinnamic acid biosynthetic process, among others. Most DEGs were associated with ribosomal biogenesis (Supplementary Figure 2). Among the processes, most GO terms were associated with DNA replication initiation, with seven terms accounting for 30.43% in the group, followed by carotenoid metabolic process and fructose 1,6-bisphosphate 1-phosphatase activity, with three and two terms accounting for 13.04 and 8.7%, respectively (Figure 2D). Ribosomes, similar to remaining processes, contained one GO term that accounted for only 4.35% (Figure 2D).

**Figure 3:**
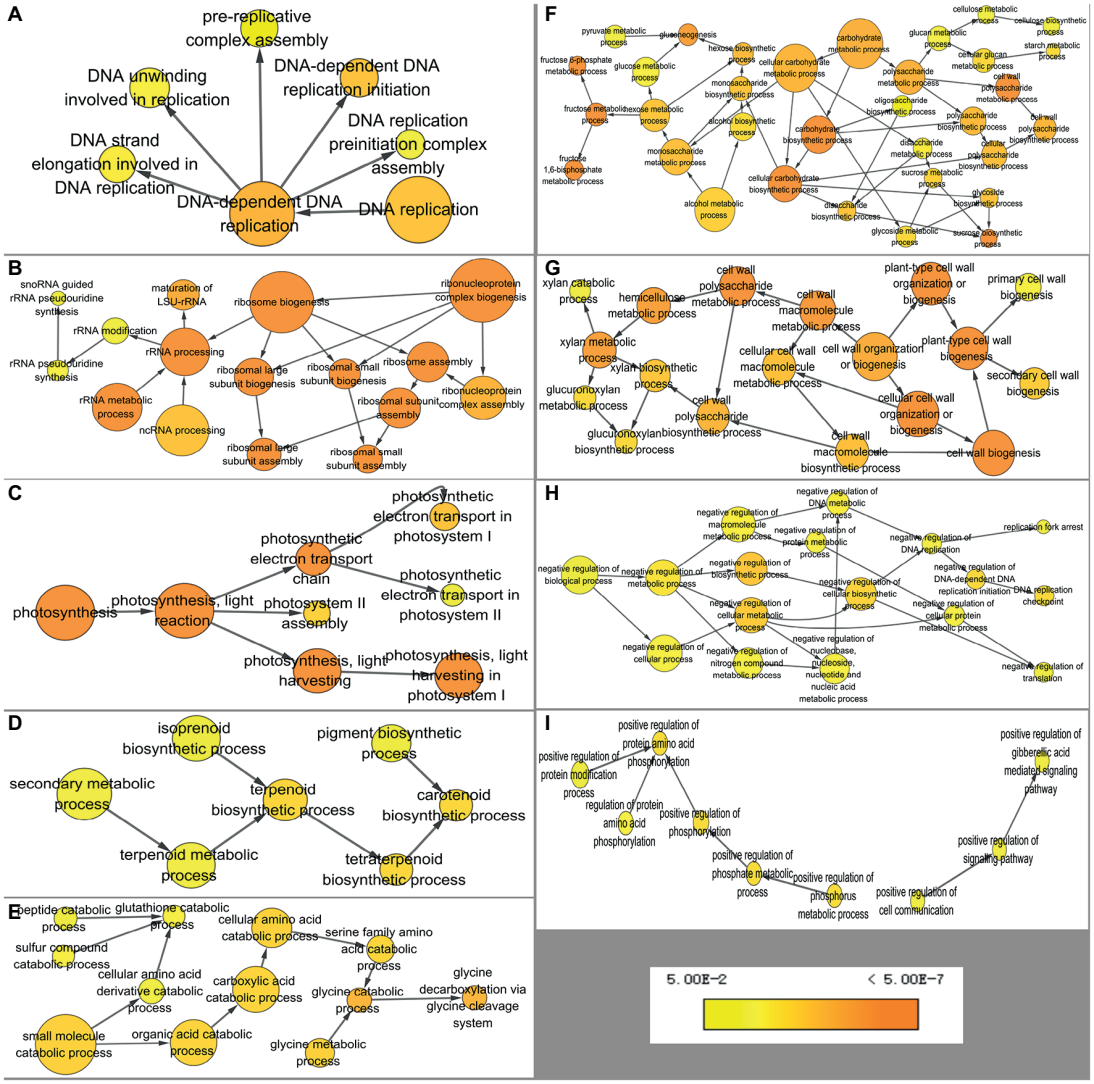
Overrepresented GO (gene ontology) terms of differentially expressed genes annotated by BiNGO separated into nine groups. **(A)** DNA replication, **(B)** ribosomal biogenesis, **(C)** photosynthesis, **(D)** carotenoid biosynthesis, **(E)** catabolism of amino acids, **(F)** carbohydrate metabolism, **(G)** cell wall organization or biogenesis, **(H)** negative regulation, and **(I)** positive regulation. Color of a node represents the corrected *p*-value, with the scale ranging from yellow (*p* = 0.01) to dark orange (*p* = 0.01 × 10^−5^) and size of a node indicates the number of genes involved.

To increase understanding of the pathways associated with DEGs, the BiNGO app in Cytoscape software was used to annotate overrepresented GO terms and present them in a network. Terms were combined into nine groups: DNA replication, ribosome biogenesis, photosynthesis, carotenoid biosynthesis, carbohydrate metabolism, cell wall organization or biogenesis, glycine decarboxylation, negative regulation of biological process, and positive regulation of biological process (Figure 3). In JcFT^OE^ plants, there were a variety of effects on DNA replication, including on preinitiation complex assembly, pre-replicative complex assembly, and DNA unwinding, strand elongation, and replication initiation, among which pre-replicative complex assembly and replication initiation were most significantly affected (Figure 3A). Ribosomal biogenesis involved maturation of the large subunit (LSU) rRNA and assembly of large and small ribosomes (Figure 3B). Effects on photosynthesis primarily included those on light harvesting and electron transport in photosystem I and II assemblies (Figure 3C). Effects on carotenoid biosynthesis involved terpenoid biosynthesis (Figure 3D). Catabolism of amino acids, primarily glycine and serine, was also affected (Figure 3E). Effects on carbohydrate metabolism included those on fructose 6-phosphate and 1,6-diphosphate metabolic processes; gluconeogenesis and the biosynthesis of sucrose; starch metabolism; and cellulose biosynthesis (Figure 3F). In cell wall organization or biogenesis, biogenesis of both primary and secondary cell walls was significantly affected, largely because of effects on glucuronoxylan biosynthesis and hemicellulose metabolism (Figure 3G). In JcFT^OE^ plants, there was both negative and positive regulation. Negative regulation primarily involved those genes associated with DNA replication (including replication fork arrest and replication checkpoints), protein metabolic process and translation, and nucleobase, nucleoside, nucleotide, and nucleic acid metabolic processes (Figure 3H). Positive regulation primarily involved those genes associated with gibberellic acid (GA)-mediated signaling pathway and protein amino acid phosphorylation (Figure 3I).

**Figure 4:**
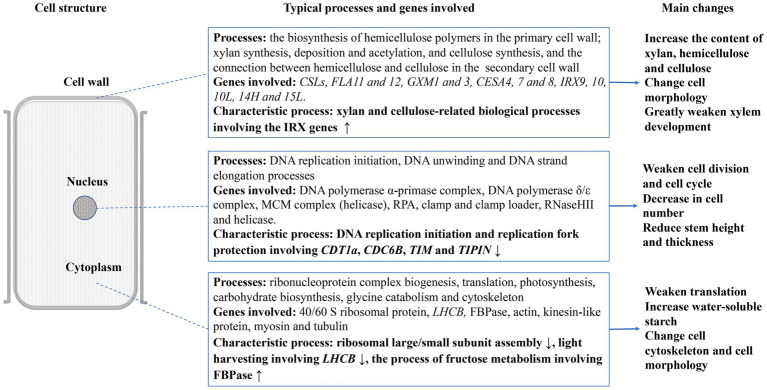
Overview of effects on stem development in JcFT^OE^ plants (↑ and ↓ denote up- and down-regulated expression, respectively).

The biological processes associated with DEGs indicated that the stem development was affected in three spatial systems in JcFT^OE^ plants. The nucleus, cytoplasm, and cell wall systems are described in Figure 4. DNA replication was the main process affected in the nucleus. In the cytoplasm, ribosome biogenesis, translation, photosynthesis, carbohydrate biosynthesis, and the cytoskeleton were primarily affected. The primarily effect in cell walls was associated with xylan (a component of hemicellulose) and cellulose.

### Effects of JcFT^OE^ on Circadian Rhythm

**Figure 5:**
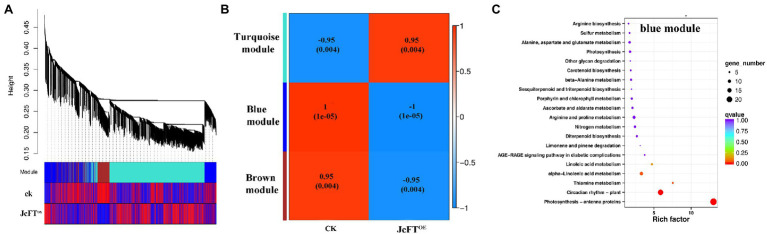
Weighted Gene Co-Expression Network Analysis (WGCNA) of differentially expressed genes (DEGs) and Kyoto Encyclopedia of Genes and Genomes (KEGG) pathway enrichment analysis of the blue module. **(A)** Dendrogram of DEGs and heat map of trait correlations. The map is divided into three parts: top, dendrogram of DEGs; middle, colors of modules of corresponding DEGs; bottom, correlations between sample DEGs and their modules. An increase in redness indicates a more positive correlation, and an increase in blueness indicates a more negative correlation. **(B)** Correlation heat map between modules and traits. The closer the correlation is to the absolute value of 1, the more likely the trait is associated with gene function of the module. **(C)** KEGG pathway enrichment analysis of genes in the blue module.

In WGCNA of this study, 6,499 of 9,564 DEGs (67.9%) clustered into three modules (Figure 5A). The turquoise module was positively correlated with JcFT^OE^ (*r* = 0.95), whereas blue and brown modules were negatively correlated with JcFT^OE^ (*r* = −1 and −0.95, respectively; Figure 5B).

The blue module had the highest correlation with JcFT^OE^, with a coefficient of −1, indicating the type of DEGs and associated biological processes in the module best reflected the influence of JcFT^OE^. In the KEGG enrichment analysis, the main biological processes were circadian rhythm and antenna proteins (Figure 5C). Circadian rhythm genes of the blue module included *late elongated hypocotyl* (*LHY*), *pseudo-response regulator 5* (*PRR5*), *timing of CAB expression 1* (*TOC1*), *gigantea* (*GI*), *early flowering 3* (*ELF3*), *phytochrome B* (*PHYB*), *constitutive photomorphogenesis 1* (*COP1*), *flavin-binding kelch domain F box protein 1* (*FKF1*), and *protein long hypocotyl 5* (*HY5*). The genes *LHY*, *TOC1*, *PRR5*, and *GI* are associated with core oscillators (Nohales and Kay, 2016), and changes in their transcription levels were greater than those of other factors. Of four *PRR5* genes, two were up-regulated (log2 FC = 2.75) and two were down-regulated (log2 FC = −3.82). Two *LHY* genes were down-regulated (log2 FC = −8.07), whereas five *TOC1* and *GI* genes were up-regulated (log2 FC = 4.98 and 2.38, respectively).

### Effects of JcFT^OE^ on DNA Replication

**Figure 6:**
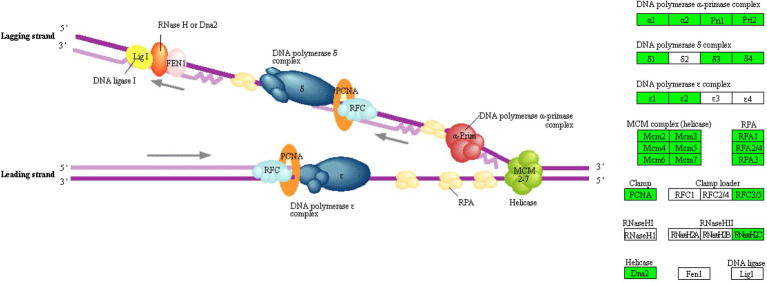
KEGG (Kyoto Encyclopedia of Genes and Genomes) pathway of DNA replication where enzymes/proteins from the DEGs (differentially expressed genes) are located (Green boxes on the right indicate DEGs with down-regulated expression, whereas white boxes indicate no change in expression).

According to results of the BiNGO analysis, in JcFT^OE^ plants, the typical effect on the nucleus was associated with DNA replication, which is also the key point that affects the cell cycle and cell division. Genomic DNA replication consists of three stages: initiation, elongation, and termination (Xue et al., 2015). In this study, 64 DEGs were associated with DNA replication (Supplementary Table 3). All of those genes were down-regulated in JcFT^OE^ tobacco stems, including those associated with DNA replication initiation, DNA unwinding, and DNA strand elongation (Figure 3A). The 64 DEGs were associated with almost all of the components required for DNA replication, including DNA polymerase α-primase complex, DNA polymerase δ/ε complex, minichromosome maintenance protein (MCM) complex, replication protein A (RPA), clamp and clamp loader, RNaseHII, and helicase (Figure 6). Therefore, the weakening of DNA replication in JcFT^OE^ plants was likely the result of comprehensive effects on the replication process.

### Effects of JcFT^OE^ on Cell Cycle Regulation and Cell Division

**Figure 7:**
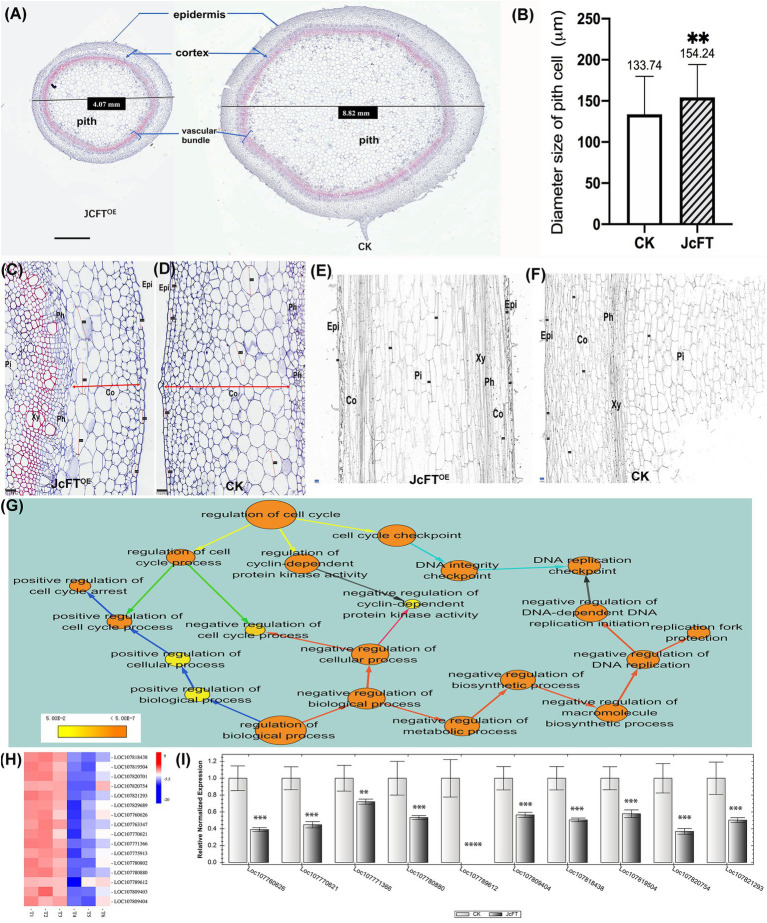
Comparison of stem cell morphology, number, and size and cell cycle regulation between JcFT^OE^ and control (CK) plants. **(A)** Stem cross sections of JcFT^OE^ and control plants (The values in the central boxes represented the diameter size of the samples, scale bar = 1 mm). **(B)** Average size of pith cells in JcFT^OE^ and control plants (The values represented mean, the vertical bars indicated standard deviation, *n* = 66, ^**^p < 0.01). **(C,D)** Stem cross sections (same magnification) of JcFT^OE^ and control plants at 49 days after seeding (scale bar = 100 μm). Double arrows indicate thickness of the cortex, and straight lines indicate larger cells in the cortex. **(E,F)** Stem longitudinal sections (same magnification) of JcFT^OE^ and control plants at 49 days after seeding (scale bar = 100 μm). **(G)** BiNGO annotated overrepresented GO (gene ontology) terms associated with cell cycle regulation. Color of a node represents the corrected *p*-value, with the scale ranging from yellow (*p* = 0.01) to dark orange (*p* = 0.01 × 10^−5^) and size of a node indicates the number of genes involved. Different colors of arrows suggested different types of enriched pathways. **(H)** Expression heat map of differentially expressed genes (DEGs) associated with DNA-dependent DNA replication initiation. Control samples: T1, T2, T3; JcFT^OE^ samples: T4, T5, T6. Blue indicates a decrease in gene expression; red indicates an increase in gene expression. **(I)** Reverse-transcription qPCR of 10 DEGs associated with DNA-dependent DNA replication initiation (bars represented gene expression mean and standard error of mean, *n* = 3, ^**^p < 0.01, ^***^p < 0.001, ^****^p < 0.0001, and the corresponding gene accession number was under the bars.). Epi: epidermis; Co: cortex; Ph: phloem; Xy: xylem; Pi: pith.

Stem diameters of JcFT^OE^ tobacco plants were significantly smaller than those of control plants (Figures 11, 7A), which was most likely due to changes in cell size or number. In JcFT^OE^ tobacco plants, pith cells, which accounted for the highest proportion of the stem, were significantly larger than those in control plants (Figure 7B). Stem epidermis and cortex cells in JcFT^OE^ plants were also larger than those in control plants. Specifically, some cortex cells were significantly longer than those in the control, resulting in elliptical cells compared with the nearly round cells in the control (Figures 7C,D). However, number of cell layers in cortex, pith, and vascular bundles of JcFT^OE^ tobacco stems decreased (Figures 7A,C,D). In longitudinal sections of JcFT^OE^ stems, although there were fewer longitudinal cells, they were also longer than those in control stems (Figures 7E,F). Those results confirmed that a significant decrease in number of cells in JcFT^OE^ stems was the primary reason for smaller and shorter stems. Changes in cell number are the result of cell division and proliferation, which are regulated by the cell cycle. Therefore, the biological processes of cell cycle regulation annotated by ClueGO were analyzed. The data set contained 55 DEGs, of which 48 were down-regulated (Supplementary Table 4).

BiNGO was used to further annotate the 55 DEGs, and in cell cycle regulation, there were three aspects: cell cycle checkpoint, regulation of cyclin-dependent protein kinase activity, and regulation of cell cycle process (yellow arrows in Figure 7G). The DEGs associated with cell cycle checkpoint were primarily DNA replication checkpoint genes (cyan arrows in Figure 7G), which were the same as those associated with negative regulation of DNA-dependent DNA replication initiation. The cell cycle process was regulated both negatively and positively (green arrows in Figure 7G), with the positive regulation primarily associated with cell cycle arrest. Furthermore, when the 55 DEGs were annotated to the biological regulation process, both negative and positive regulation were also detected. Negative regulation was primarily associated with DNA replication in DNA-dependent DNA replication initiation and replication fork protection and cell cycle process and cyclin-dependent protein kinase activity (red arrows in Figure 7G). Positive regulation was associated with cell cycle arrest (blue arrows in Figure 7G). The analyses suggested that the cell cycle was inhibited in JcFT^OE^ plants.

According to the above BiNGO annotation, negative regulation of DNA replication was the most important effect on the cell cycle in JcFT^OE^ plants. The DNA-dependent DNA replication initiation was the key process and was associated with 16 genes, which primarily encoded CDT1-like protein A (CDT1a) and cell division control protein 6 (CDC6) homolog B. The genes were all down-regulated in JcFT^OE^ stems (Figure 7H). Expression levels of 10 genes analyzed by RT-qPCR (Figure 7I) were consistent with those of the transcriptome analysis. The proteins CDT1 and CDC6 have key roles in regulating DNA replication as well as in activation and maintenance of cell cycle checkpoints (Truong and Wu, 2011; Youn et al., 2020). Down-regulation of CDT1 in *Arabidopsis thaliana* alters both nuclear DNA replication and plastid division, slowing the cell cycle and resulting in smaller leaves than those of wild-type plants (Raynaud et al., 2005).

Replication fork protection was associated with five down-regulated timeless (TIM) and timeless-interacting proteins (TIPIN). During DNA replication, the TIM–TIPIN heterodimer forms a replication fork protection complex (FPC). The FPC is associated and moves with the replication fork to help maintain its integrity and stability, thereby ensuring an effective replication process (Errico and Costanzo, 2012; Rageul et al., 2020; Lo et al., 2021). Positive regulation of cell cycle arrest was associated with four down-regulated breast cancer-associated gene 1 (BRCA1) and BRCA1-associated RING domain protein 1 (BARD1), which are components of replication fork protection (Daza-Martin et al., 2019). Therefore, results in this study suggested that down-regulation of TIM, TIPIN, BRCA1, and BARD1 reduced replication efficiency.

Down-regulation of CDT1, CDC6, TIM, TIPIN, MCM, BRCA1, and BARD1 in JcFT^OE^ plants slowed formation of the pre-replication complex and the replication fork protection complex, thereby reducing the efficiency of replication initiation and strand elongation. Because of the decrease in DNA replication efficiency and cell division, numbers of cells in stems decreased significantly. Combined with the transcriptome data, these results suggested that the decrease in cell number was the key reason why stem diameters decreased in JcFT^OE^ plants.

### Effects of JcFT^OE^ on Ribonucleoprotein Complex Biogenesis, Photosynthesis, Carbohydrate Biosynthesis, and Cytoskeleton

In the cytoplasm of JcFT^OE^ plants, the biological processes primarily affected included ribonucleoprotein complex biogenesis, translation, photosynthesis, and carbohydrate biosynthesis. The cytoskeleton was also affected.

Ribonucleoprotein complex biogenesis was associated with 209 DEGs, of which 178 were down-regulated and 31 were up-regulated in JcFT^OE^ tobacco stems. According to BiNGO annotations, the 209 DEGs were primarily associated with ribosomal small/large subunit assembly and rRNA processing (Figure 3B), and their expression was significantly down-regulated. Down-regulated genes included 40 of 43 associated with ribosomal large subunit assembly, 27 of 29 associated with ribosomal small subunit assembly, and 84 of 110 associated with rRNA processing.

Effects on photosynthesis were largely reflected in light harvesting in photosystem I and electron transport chain of the light reaction (Figure 3C). Light harvesting was associated with 35 DEGs, all of which were *light-harvesting chlorophyll a/b binding protein* (*LHCB*) genes, with 30 that were down-regulated and five that were up-regulated. The LHCB proteins typically form a complex with chlorophyll and xanthophylls that serves as the antenna complex (Pietrzykowska et al., 2014). Gene expression of *LHCB* is regulated by multiple environmental and developmental cues, which primarily include light, chloroplast retrograde signal, the circadian clock, and the phytohormone abscisic acid (ABA; Xu et al., 2012). Therefore, in JcFT^OE^ plants, stem development was affected by the down-regulation of many *light-harvesting chlorophyll a/b binding* genes in photosystem I, which might weaken light-harvesting ability. However, the electron transport chain contained 16 genes (of which 14 were up-regulated) that functioned in hydrolysis, redox, ATP, and NDH production, with encoded proteins including oxygen-evolving enhancer protein 3 (PsbQ), PGR5-like protein 1A (PGRL1), photosynthetic NDH subunit of lumenal location (PNSL2), and ATP synthase subunit delta.

**Figure 8:**
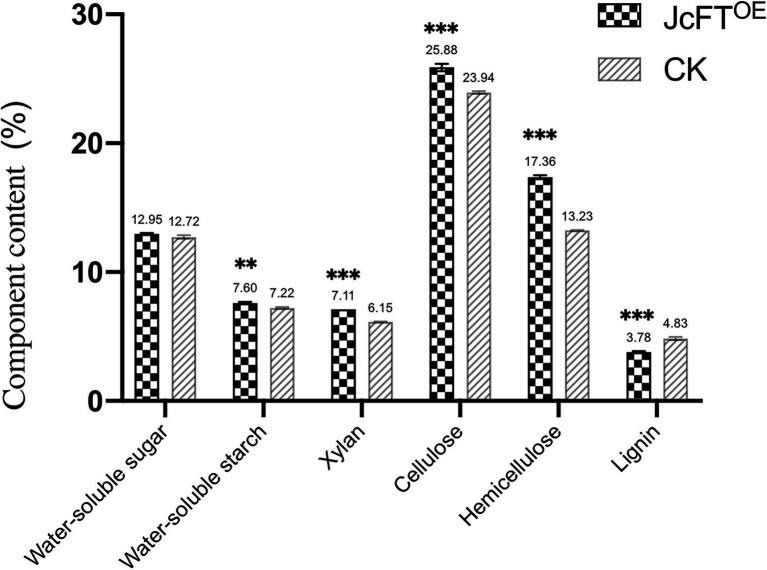
Carbohydrate components in tobacco stems of JcFT^OE^ and control (CK) plants (The values represented mean, the vertical bars indicated standard deviation, *n* = 3, ^**^p < 0.01; ^***^p < 0.001).

Carbohydrate biosynthesis included fructose metabolism, gluconeogenesis, and polysaccharide metabolism (Figure 3F). Fructose metabolism was the most important process and was associated with 26 DEGs. Twenty-three of those DEGs were up-regulated, with encoded proteins including fructose 1,6-bisphosphatase, fructose 2,6-bisphosphatase, fructose-bisphosphate aldolase, and fructose kinase-2. The DEGs associated with sucrose metabolism were all fructose metabolism-related genes (Supplementary Table 5). However, the DEGs associated with gluconeogenesis metabolism included genes other than those associated with fructose metabolism. Therefore, it was hypothesized that changes in fructose metabolism might subsequently cause changes in gluconeogenesis metabolism. Among the DEGs associated with polysaccharide metabolism, eight up-regulated genes were associated with starch biosynthesis, with four encoding 1,4-alpha-glucan-branching enzyme 1 and 4 encoding phosphoglucan phosphatase. Therefore, it was hypothesized that the starch content of JcFT^OE^ tobacco stems would be higher than that in control stems. Indeed, water-soluble starch content of JcFT^OE^ tobacco stems was significantly higher than that of control stems, whereas water-soluble sugar content was not significantly different (Figure 8).

According to the KOG classification, 70 DEGs were closely associated with the cytoskeleton (Figure 2A and Supplementary Table 6), suggesting strong effects on cell cytoskeletons in stems of JcFT^OE^ plants. Major types of genes encoded actin, tubulin, kinesin-like protein, and myosin, with the greatest number of genes encoding kinesin-like proteins. The different types of cytoskeleton-related genes were both up- and down-regulated. The cytoskeleton is a dynamically adaptive structure composed of microtubules and actin filaments (Lian et al., 2021). Microtubules are dynamic heteropolymers of α and β-tubulin that coordinate their assembly in response to various intracellular and extracellular signals and have key roles in the cell cycle and cell wall construction (Verma, 2001; Cai, 2010). The actin cytoskeleton has a key role in many cellular processes that regulate cell growth and morphology (Hussey et al., 2006; Breuer et al., 2017). Kinesins and myosins are motor proteins that actively move along microtubules and actin filaments, respectively, and perform transport functions (Nakamura et al., 2014; Nebenfuhr and Dixit, 2018). Therefore, changes in expression of genes associated with the cytoskeleton of JcFT^OE^ tobacco stems were coordinated with changes in cell division and cell morphology.

### Effects of JcFT^OE^ on Cell Wall

Plant cell walls are composed primarily of cellulose, pectins, hemicelluloses, and lignin (Rennie and Scheller, 2014). Hemicelluloses include xyloglucans, xylans, mannans, glucomannans, and β-(1 → 3,1 → 4)-glucans (Scheller and Ulvskov, 2010). In JcFT^OE^ plants, primary and secondary cell wall biogenesis were both affected, primarily by affecting hemicellulose metabolism (Figure 3G).

Biogenesis of the primary cell wall was associated with 10 DEGs. Seven up-regulated genes encoded cellulose synthase-like proteins E1, E6 (CSLE1, CSLE6), G2, and G3 (CSLG2, CSLG3), and three down-regulated genes encoded cellulose synthase-like protein D3 (CSLD3). The genes were all *cellulose synthase-like* (*CSL*) genes that encoded glycosyltransferases, which were likely associated with hemicellulose polymer biosynthesis (Galinousky et al., 2020).

Thirty-two DEGs were associated with secondary cell wall biogenesis. Sixteen of those were annotated as *glucuronoxylan 4-O-methyltransferase* (*GXM*), *β-1,4-xylosyltransferase* (associated with *irregular xylem* (*IRX*) *9*, *10*, *10-like*, and *14H*), and *IRX15-like* genes. Seven were annotated as *cellulose synthase A catalytic subunit 4*, *7*, *8* (*CESA4*, *7*, *8*) genes, and seven were annotated as *fasciclin-like arabinogalactan 11* or *12* (*FLA11* or *FLA12*) genes. The two other genes were annotated as *wall acetylation 3* (*RWA3*) and *trichome birefringence-like 16* (*TBL16*). Except for one down-regulated gene (*TBL16*), the other 31 genes were up-regulated. Furthermore, among the 32 DEGs associated with secondary cell walls, 18 were associated with xylan biosynthesis, deposition, and acetylation, whereas the others were associated with cellulose biosynthesis and the connection between hemicellulose and cellulose.

In addition, 36 DEGs associated with hemicellulose metabolism were all xylan metabolism-related proteins. According to Swiss-Prot annotations, the genes primarily encoded beta-D-xylosidase, glucuronoxylan 4-O-methyltransferase, beta-1,4-xylosyltransferase, xylan glucuronosyltransferase, and protein IRX15-like. Those genes associated with xylan biosynthesis, with one exception, were all up-regulated in JcFT^OE^ stems (Supplementary Table 7). Therefore, in JcFT^OE^ plants, hemicellulose was affected by increases in xylan biosynthesis. In the analysis of stem carbohydrate contents, xylan and hemicellulose contents in JcFT^OE^ tobacco stems were indeed significantly higher than those in the control (Figure 8).

In summary, stem development in JcFT^OE^ tobacco plants was affected by increases in contents of xylan, hemicellulose, and cellulose in cell walls.

### Effects of JcFT^OE^ on Vascular Bundle Development

**Figure 9:**
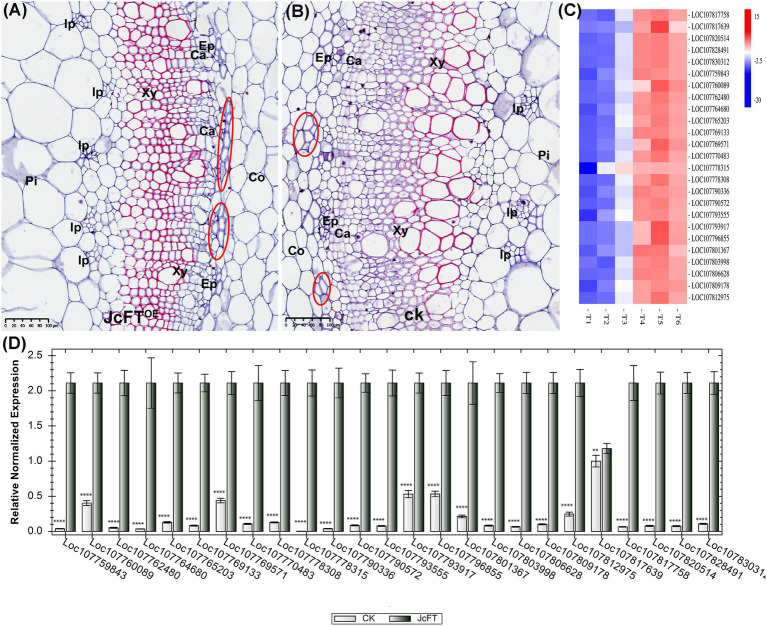
Development of stem vascular bundles in JcFT^OE^ and control plants. **(A,B)** Stem cross sections (same magnification) of JcFT^OE^ and control plants at 49 days after seeding (scale bar = 50 μm). Whereas JcFT^OE^ plants have five smaller internal phloem bundles, control plants have three larger internal phloem bundles. Circles indicate fiber cells in the external phloem. **(C)** Expression heat map of 25 differentially expressed genes identified as *IRX* genes. Control samples: T1, T2, T3; JcFT^OE^ samples: T4, T5, T6. Blue indicates a decrease in expression; red indicates an increase in expression. **(D)** Reverse-transcription qPCR verification of 25 differentially expressed *IRX* genes (bars represented gene expression mean and standard error of mean, *n* = 3, ^**^p < 0.01, ^****^p < 0.0001, and the corresponding gene accession number was under the bars). CK: control; Pi: pith, Co: cortex, Ph: phloem, Ca: cambium, Xy: xylem, Ip: internal phloem, Ep: external phloem.

Tobacco stems contain bicollateral vascular bundles (Boucheron et al., 2002). The stele has six parts from outside to inside: pericycle, external phloem, cambium, xylem, internal phloem, and pith. In stem sections, morphology and structure of the stem vascular bundle in JcFT^OE^ plants were clearly different compared with those of control plants. Stems of JcFT^OE^ plants had more internal phloem bundles than those of controls, and the external phloem had stronger phloem fibers. In addition, JcFT^OE^ stems had fewer cambium cells than those of control stems, indicating weaker secondary growth. In particular, the degree of xylem development in JcFT^OE^ tobacco was significantly weaker than that in control tobacco and was characterized by fewer cell layers, weaker cell division and differentiation, and smaller vessels (Figures 9A,B).

Thirty-two DEGs were associated with secondary cell wall biogenesis. According to previous studies (Zang et al., 2015; Zhong et al., 2019), 25 of the DEGs were *IRX* genes (Supplementary Table 8). The *IRX* genes are closely associated with xylem development and encode enzymes or transcription factors that participate in biosynthesis of secondary wall cellulose, xylan, lignin, and pectin (Hao and Mohnen, 2014). In this study, all *IRX* genes were up-regulated (Figures 9C,D), with 13 associated with xylan biosynthesis, seven with cellulose biosynthesis, and five with microfibril orientation. Thus, the up-regulated DEGs contributed to increasing contents of xylan (a component of hemicellulose) and cellulose in the xylem. According to the transcription data, there were no differences in lignin-related gene expression. Therefore, contents and characteristics of hemicellulose and cellulose in secondary cell walls might be the primary factors affecting xylem development in JcFT^OE^ plants. Moreover, such increases in cell wall xylan and cellulose contents might weaken xylem differentiation and development.

### Effects of JcFT^OE^ on Plant Hormones

Florigen is a proteinaceous hormone that has wide-ranging regulatory effects on growth (Lifschitz et al., 2014). In addition to inducing flowering, it also regulates, for example, tuber formation in potatoes, leaf size in Arabidopsis and tobacco, cluster shape in grape, and bud formation in poplar (Danilevskaya et al., 2011; Shalit-Kaneh et al., 2019). In this study, signal transduction of a variety of plant hormones was affected in JcFT^OE^ plants, including auxin, cytokinin, GA, ABA, and salicylic acid (SA). Therefore, the FT protein hormone may interfere with functions of a variety of other plant hormones by affecting hormone signal transduction.

**Figure 10:**
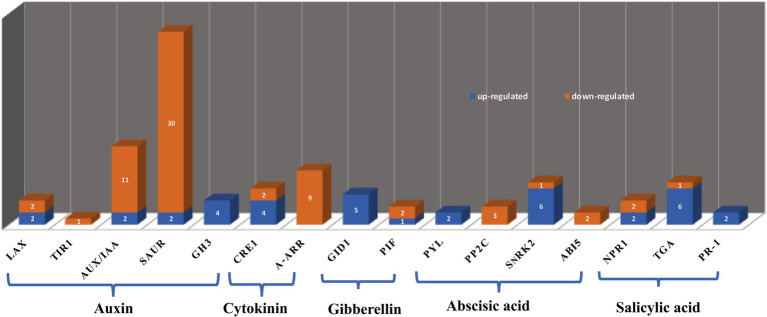
Hormone types and associated differentially expressed genes (DEGs). The *y*-axis indicates number of DEGs displayed on the bars, and the *x*-axis shows names of DEGs. LAX: like-aux1; TIR1: transport inhibitor response protein 1; Aux/IAA: including auxin/indole-3-acetic acid; SAUR: small auxin up RNA; GH3: gretchen hagen 3; CRE1: cytokinin receptor 1; A-ARR: type-A authentic response regulator; GID1: gibberellin-insensitive dwarf 1; PIF: phytochrome-interacting factor; PYL: pyrabactin resistance 1-like; PP2C: clade A protein phosphatases type-2C; SnRK2: sucrose non-fermenting 1-related subfamily 2; ABI5: abscisic acid-insensitive 5; NPR1: nonexpresser of PR gene 1; TGA: TGACG sequence-specific binding proteins; PR-1: pathogenesis-related protein 1.

Transcriptome data showed that the JcFT florigen protein had different effects on different hormone signal transduction pathways. The primary factors associated with each pathway and numbers of up- and down-regulated DEGs are presented in Figure 10. The greatest number of DEGs was associated with the auxin signal transduction pathway. Differentially expressed genes associated with auxin and cytokinin tended to be down-regulated in JcFT^OE^ stems, whereas those associated with gibberellin, ABA, and SA were up-regulated.

There were three major families of auxin early response genes, including *auxin/indole-3-acetic acid* (*Aux/IAA*), *gretchen hagen 3* (*GH3*), and *small auxin up RNA* (*SAUR*). Most DEGs were *SAUR* and *Aux/IAA* genes, and most were down-regulated. However, *GH3* was up-regulated in JcFT^OE^ stems. In addition, *auxin influx carriers like-aux1* (*LAX*) and *transport inhibitor response protein 1* (*TIR1*) were generally down-regulated. The largest family of early auxin-responsive genes in higher plants is the *SAUR* family, but the function of only a few *SAUR* genes is known (Zhang et al., 2021). *SAUR* genes can affect the distribution of indole-3-acetic acid (IAA; Huang et al., 2020). For example, *SAUR69* inhibits auxin transport in tomato fruits (Shin et al., 2019), and *SAUR45* affects auxin biosynthesis and transport in rice (Xu et al., 2017). In this study, *SAUR* was primarily associated with auxin-induced protein 15A/15A-like. Therefore, the function of many of the *SAUR* DEGs in this study might be associated with transport and biosynthesis of auxin. However, further research is needed. The *GH3* gene encodes an enzyme that catalyzes the coupling of free IAA to amino acids and therefore primarily regulates growth and development by regulating the level of free IAA. Overexpression of *GH3* in rice leads to a decrease in free IAA content and dwarfing of transgenic plants (Gan et al., 2019). In this study, four *GH3*s were all up-regulated, suggesting free IAA levels decrease in JcFT^OE^ plants. Aux/IAA family members may bind with auxin response factors (ARFs) and repress expression of genes activated by ARFs in the absence of auxin. When Aux/IAA is degraded by the 26S proteasome to release ARFs at high auxin levels, auxin response genes are expressed (Luo et al., 2018). In this study, down-regulation of Aux/IAA in JcFT^OE^ tobacco stems indicated that the amount of bound ARF decreased. The auxin influx carrier identified in this study was LAX2, which is a functional auxin influx carrier implicated in regulating vascular development in cotyledons (Swarup and Peret, 2012).

Thus, in JcFT^OE^ in tobacco stems, biosynthesis and transport of auxin and content of free IAA might decrease, which would regulate expression of auxin response genes. Auxin is essential in formation of the vascular system, and it has an important regulatory role in early transdifferentiation into xylem cells (Yoshida et al., 2009). Therefore, changes in auxin signaling pathway factors might be one of the most significant factors that led to changes in vascular bundles of JcFT^OE^ tobacco plants.

In the cytokinin signaling pathway of JcFT^OE^ plants, effects were generally associated with cytokinin receptor 1 (CRE1) and type-A authentic response regulator (ARR). Of type-A ARRs, ARR 5, 6, 9, and 15 were all down-regulated in JcFT^OE^ tobacco. The genes encoding those proteins are negative regulators of cytokinin response, and their mutants have increased sensitivity to cytokinins (To et al., 2004; Bhaskar et al., 2021). Therefore, stems in JcFT^OE^ tobacco might be more sensitive to cytokinins than those of the control.

The gibberellin signaling pathway was primarily associated with GID1 and phytochrome-interacting factor 3, 5 (PIF3, 5), with GID1 the most important in the positive regulation of gibberellin signals described above.

The ABA signal transduction pathway was associated with pyrabactin resistance 1-like (PYL), clade A protein phosphatases type-2C (PP2C), sucrose non-fermenting 1-related subfamily 2 (SnRK2), and ABA-insensitive 5 (ABI5). The PYL receptors have major roles in ABA sensing and signal transduction. They perceive intracellular ABA and form a ternary complex with PP2Cs, thereby inhibiting them. The inhibition allows activation of downstream targets of PP2Cs, including SnRK2 protein kinase, which has a key role in regulation of the transcriptional response to ABA (Bueso et al., 2014). The most PYL and SnRK2 in JcFT^OE^ tobacco stems were up-regulated, whereas most PP2Cs were down-regulated. Therefore, the response to ABA likely increased in JcFT^OE^ stems.

The SA signaling pathway was associated with nonexpresser of PR gene 1 (NPR1), TGACG sequence-specific binding proteins (TGA), and pathogenesis-related (PR) protein 1. Notably, the six associated TGA 1 or 2 and two PR1 proteins were all up-regulated in JcFT^OE^ tobacco stems. Expression of *PR* genes is associated with induction of plant systemic acquired resistance (Durrant and Dong, 2004). Salicylic acid activates defense responses through its downstream component NPR1 (Zhang et al., 2010). The TGA transcription factors regulate *PR* genes because they physically interact with the known positive regulator NPR1 (Kesarwani et al., 2007). Therefore some aspects of tobacco resistance might also increase in JcFT^OE^ plants. Repeated observations indicate that JcFT^OE^ tobacco is more resistant to plant hoppers than the control, because under the same conditions, control tobacco leaves wilted under plant hopper attack, whereas JcFT^OE^ tobacco leaves remained firm and healthy (data not shown).

## Discussion

In the JcFT^OE^ plants, stem stature and thickness decreased. Stems of most plants that overexpress *FT* or *FT-like* genes are similarly affected (Lifschitz et al., 2006; Li et al., 2015; Gao et al., 2016; Adeyemo et al., 2017; Pasriga et al., 2019; Odipio et al., 2020). Therefore, inhibition of stem growth is likely a basic effect of the *FT* gene. However, the mechanisms by which *FT* causes stem thinning have not been fully investigated. The results in this study indicated that stem thinning might be due to the slowing of cell division because of effects on DNA replication and the cell cycle, which ultimately decreased cell number and resulted in thinner and shorter stems. Shalit et al. (2009) investigated the tomato precursor of florigen, single flower truss (SFT), and a potent SFT-dependent SFT inhibitor, self-pruning (SP). They found that a high SFT/SP ratio is associated with growth restriction of the shoot apical meristem, which resulted in faster transformation to flowering (Shalit et al., 2009). In this study, an *SP* (LOC107810240) was significantly down-regulated (log2 FC = −4.83) in JcFT^OE^ tobacco stems, therefore, *FT* could reduce expression of the *SP* gene in stems, further increasing the FT/SP ratio. We speculate that the transition from vegetative to reproductive growth may involve short-term slowing of growth in order to complete the transition. The inhibitory function of *FT* on stem cell division demonstrated in this study might be the trigger for such a slowdown.

In the WGCNA, DEGs associated with circadian rhythm and antenna proteins were grouped into a module that was most highly correlated with JcFT^OE^ (*r* = −1; Figure 5C). Several *cis*-acting sequence elements have been identified for circadian control of *CAB* gene expression (Millar and Kay, 1996; Andronis et al., 2008). Transcription of *CAB* genes is circadian-regulated (Millar and Kay, 1991), and rhythmic expression of *CAB* genes has often been used as a marker for circadian regulation in plants (Thain et al., 2002). Therefore, the significant changes in *CAB* expression in JcFT^OE^ plants confirmed that the circadian clock was altered.

Considering the effects of JcFT^OE^ on photosynthesis, *LHCB* was found to be mainly down-regulated, while 14 genes involved in the electron transport chain were up-regulated. These included *PGR5-like protein 1A* (*PGRL1*) and *oxygen-evolving enhancer protein 3* (*PsbQ*). The antenna complex absorbs sunlight and transfers the excitation energy for photosynthesis in green plants, and the LHCB proteins are important components of the antenna complex (Bassi et al., 1999). Studies have demonstrated the importance of the cyclic electron transport (CET)-dependent proton motive force (pmf) under low light for ATP synthesis (Walker et al., 2014). PGR5/PGRL1 and NDH can mediate CET processes in low light and facilitate CO_2_ assimilation by supplying additional ATP (Ma et al., 2021). Studies have also indicated that the PsbQ protein is required for photoautotrophic growth under low-light conditions (Yi et al., 2006). Therefore, we speculated that the down-regulated expression of *LHCB* observed in this study implied an impairment of light absorption, which may be perceived by JcFT^OE^ tobacco as a low-light signal. The up-regulated expression of the related genes in the electron transport chain observed in this study may be a synergistic response to the down-regulation of *LHCB*.

Complex effects on multiple circadian clock elements and DNA replication were also observed in JcFT^OE^ plants. Transcription levels of core oscillators of circadian rhythm (LHY, TOC1, PRR5, and GI) were significantly affected by the overexpression of *JcFT*. The core oscillator GI is a unique plant protein that is involved in many developmental processes, including flowering time regulation, circadian rhythm control, sucrose signaling, and starch accumulation, among others (Mishra and Panigrahi, 2015; Cha et al., 2017). The results suggested that changes in the circadian rhythm was a characteristic effect of *JcFT*.

Formation of a pre-replication complex is key to the control of DNA replication before a cell enters the S phase. The complex is assembled at the replication origin by sequential association of the origin recognition complex, followed by CDT1 and CDC6, which ultimately recruit the DNA helicase MCM to open the replication fork, allowing DNA replication to begin (Takisawa et al., 2000; DePamphilis, 2003; Brasil et al., 2017). Therefore, CDC6 has a key role in regulating DNA replication, as well as in activation and maintenance of cell cycle checkpoints (Youn et al., 2020). Binding of TOC1 to the *CDC6* promoter is responsible for diurnal suppression of DNA replication. Thus, TOC1 safeguards the transition from G1 to S phases and controls the timing of the early mitotic cycle and plant growth. When *TOC1* is overexpressed, plants have a shorter and delayed S phase (Fung-Uceda et al., 2018). The protein PRR5 directly down-regulates *CCA1* and *LHY* expression (Nakamichi et al., 2010), and LHY is a MYB transcription factor that directly binds to the *TOC1* promoter to negatively regulate its expression (Gendron et al., 2012). Therefore, the down-regulation of *LHY* and the up-regulation of *TOC1* in this study were generally consistent with the weaker growth of JcFT^OE^ tobacco. Changes in the core oscillator proteins LHY, PRR5, TOC1, and GI, as well as the significantly down-regulated expression of CAB antennae proteins, are among the most common effects on stems in JcFT^OE^ tobacco.

The rice FT homolog Hd3a interacts with 14-3-3 proteins, and two Hd3a monomers bind to C-terminal regions of dimeric 14-3-3 proteins to produce a complex (Taoka et al., 2011, 2013; Putterill and Varkonyi-Gasic, 2016). The 14-3-3 proteins are notable for their ability to bind a variety of signal proteins with diverse functions, including kinases, phosphatases, and transmembrane receptors. As a result, they have important roles in a wide range of important regulatory processes, including cell cycle control, mitotic signal transduction, and apoptotic cell death (Fu et al., 2000). Ligands of 14-3-3 proteins share a common binding determinant, which mediates contact with 14-3-3 proteins (Fu et al., 2000). For example, in shoot apical meristems of rice, rice centroradialis (RCN) competes with Hd3a in binding to 14-3-3 proteins and represses florigenic activity. When RCN is knocked out, Hd3a more easily binds to 14-3-3 proteins to form an excess of complexes (Kaneko-Suzuki et al., 2018). This type of interaction helps to understand why multiple biological processes were affected during stem development in JcFT^OE^ plants. Binding of the JcFT protein to 14-3-3 proteins might affect the binding of many other proteins to 14-3-3 proteins and lead to changes in expression of related genes, thereby altering many biological processes. This study demonstrated that 14-3-3 proteins could directly interact with 294 proteins of DEGs involved in 15 biological processes (Supplementary Table 9). In addition, most of the biological processes annotated from the DEGs were directly associated with 14-3-3 proteins. Therefore, 14-3-3 proteins likely have important roles in FT function. In addition, 14-3-3 proteins might be primary mediators of the effects on stem development in JcFT^OE^ tobacco.

**Figure 11:**
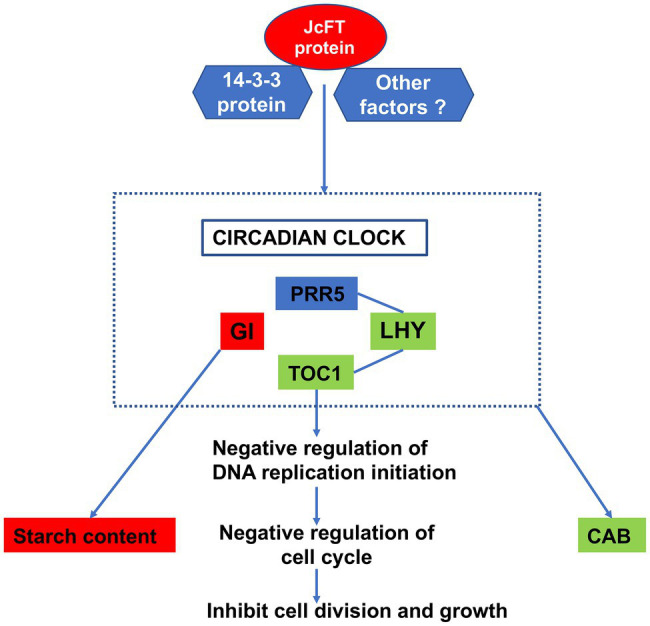
Conceptual model of possible regulation of the circadian clock and DNA replication in stems of JcFT^OE^ plants. Green boxes indicate down-regulation, red ones indicate up-regulation, and the blue one indicates both up- and down-regulation. Arrows indicate order of regulation.

The 14-3-3 proteins also mediate circadian regulation (Prado et al., 2019). Therefore, it was hypothesized that FT mediated circadian regulation *via* 14-3-3 proteins and affected DNA replication, expression of *CAB*, and other processes. A conceptual model of regulation of DNA replication and circadian rhythm developed for JcFT^OE^ plants is shown in Figure 11.

In this study, among the DEGs associated with secondary cell wall biogenesis, all 18 genes associated with xylan biosynthesis, deposition, and acetylation were up-regulated. Those genes included *glucuronoxylan 4-O-methyltransferase*, *β-1,4-xylosyltransferase*, *IRX15-like*, *CESA4*, *7*, and *8*, and *FLA*. *Glucuronoxylan 4-O-methyltransferase*, *β-1,4-xylosyltransferase*, and *IRX15-like* are closely associated with biosynthesis of glucuronoxylan, and mutations in those genes lead to decreases in xylan content (Tanaka et al., 2003; Wu et al., 2009; Mortimer et al., 2010; Brown et al., 2011). The genes *CESA4*, *7*, and *8* have essential roles in cellulose biosynthesis in secondary cell walls (Chen et al., 2005; Stork et al., 2010; McFarlane et al., 2014). A causal relationship between *FLA* transcript abundance in plant stems and cellulose microfibril orientation and wood properties has been previously reported (MacMillan et al., 2010). Because xylan is an important component of hemicellulose (Pauly et al., 2013), up-regulation of associated genes may affect the content and characteristics of hemicellulose and thereby affect cell wall development (Grantham et al., 2017). Therefore, in JcFT^OE^ plants, stem development might be affected by increases in hemicellulose and cellulose contents in secondary cell walls.

In JcFT^OE^ plants, expression of many *IRX* genes was significantly up-regulated, and as a result, xylem development was significantly affected. Clarifying how JcFT regulates *IRX* gene expression may help to understand the evolution and function of FT.

In addition to observations of stem anatomy in horizontal and longitudinal sections, epidermal cells of leaves, petioles, and stems were also examined. In JcFT^OE^ plants, an important feature was longer cells (Supplementary Figure 3). Gibberellic acid regulates various developmental processes, including elongation of cells and shoots, transition to flowering, and flower growth, among other processes (Illouz-Eliaz et al., 2019). In this study, the GA-mediated signal pathway was significantly affected in JcFT^OE^ plants (Figures 3I, 10), which might increase the response of tobacco stems to gibberellin. Therefore, it was hypothesized that the lengthening of cells in JcFT^OE^ plants was most likely caused by changes in the gibberellin signaling pathway.

In addition, it is necessary to investigate the effects of different FT overexpression levels on plant development to better understand the mechanisms through which FT regulation influences flowering and growth. We examined the effects of JcFT overexpression on flowering 37 and 42 days after sowing (Supplementary Figure 5A). The results showed significantly higher expression of JcFT in JcFT^OE^ plants that flowered at 37 days compared with those that flowered at 38–42 days. However, there was no significant difference in the expression level of JcFT in JcFT^OE^ plants flowering between 38 and 41 days (Supplementary Figure 5B). This suggests JcFT^OE^ plants with high levels of JcFT bloomed earlier. However, there was no linear relationship between the flowering sequence and the JcFT expression level. It is possible that this investigation was limited by the experimental material. The stems and leaves of the JcFT^OE^ plants used in the experiments did not differ significantly in size, and the effect of JcFT expression on stem and leaf growth could not be adequately determined. To gain a deeper understanding of the role of JcFT in development, it would be necessary to use larger samples and, especially, to develop JcFT^OE^ plants with visible differences in the stem and leaf sizes.

## Data Availability Statement

The datasets presented in this study can be found in online repositories. The names of the repository/repositories and accession number(s) can be found in the article/Supplementary Material.

## Author Contributions

JW contributed to conception and design of the study, writing of the manuscript, and experimental protocol. QW performed transcriptome analysis, RT-PCR, and statistical analysis and wrote sections of the manuscript. ZB prepared tissue sections, measured stem components, and collected experimental data. XZ contributed to the revision of the manuscript. JZ performed transgenic plant cultivation, management, and measurement. QL and WK carried out genetic transformation of tobacco and identification of transgenic plants. All authors contributed to the article and approved the submitted version.

## Funding

This research work was funded by the National Natural Science Foundation of China (31270359) and the Department of Science and Technology of Sichuan Province (2018NFP0035).

## Conflict of Interest

The authors declare that the research was conducted in the absence of any commercial or financial relationships that could be construed as a potential conflict of interest.

## Publisher’s Note

All claims expressed in this article are solely those of the authors and do not necessarily represent those of their affiliated organizations, or those of the publisher, the editors and the reviewers. Any product that may be evaluated in this article, or claim that may be made by its manufacturer, is not guaranteed or endorsed by the publisher.

## Supplementary Material

The Supplementary Material for this article can be found online at: https://www.frontiersin.org/articles/10.3389/fpls.2022.922919/full#supplementary-material

## Footnotes

^1^ https://www.ncbi.nlm.nih.gov/bioproject/208209
