# Supplementary material for: Comprehensive Effects of Flowering Locus T-Mediated Stem Growth in Tobacco
Source: Front Plant Sci. 2022 Jun 16;13:922919. doi: 10.3389/fpls.2022.922919 (PMC9243646; doi:10.3389/fpls.2022.922919)
Supplement: Supplementary file 1 [file Data_Sheet_1.ZIP › Supplementary_Material.docx]

Supplementary Material

# Supplementary Figures


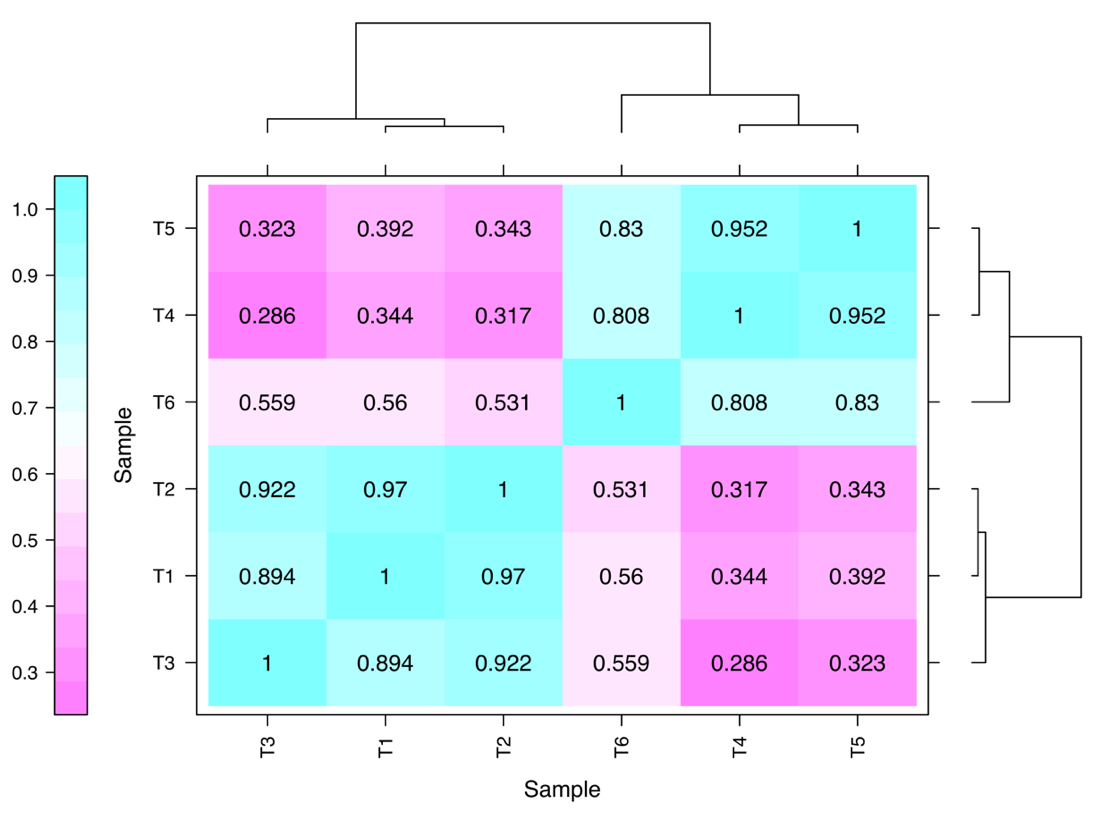


**Supplementary Figure 1.** Pairwise correlation of the expression levels of 6 samples. (The number in the middle of the chart is the pearson correlation coefficient R, and T1, T2, T3 are control samples, T4, T5, T6 are JcFT^OE^ samples.)


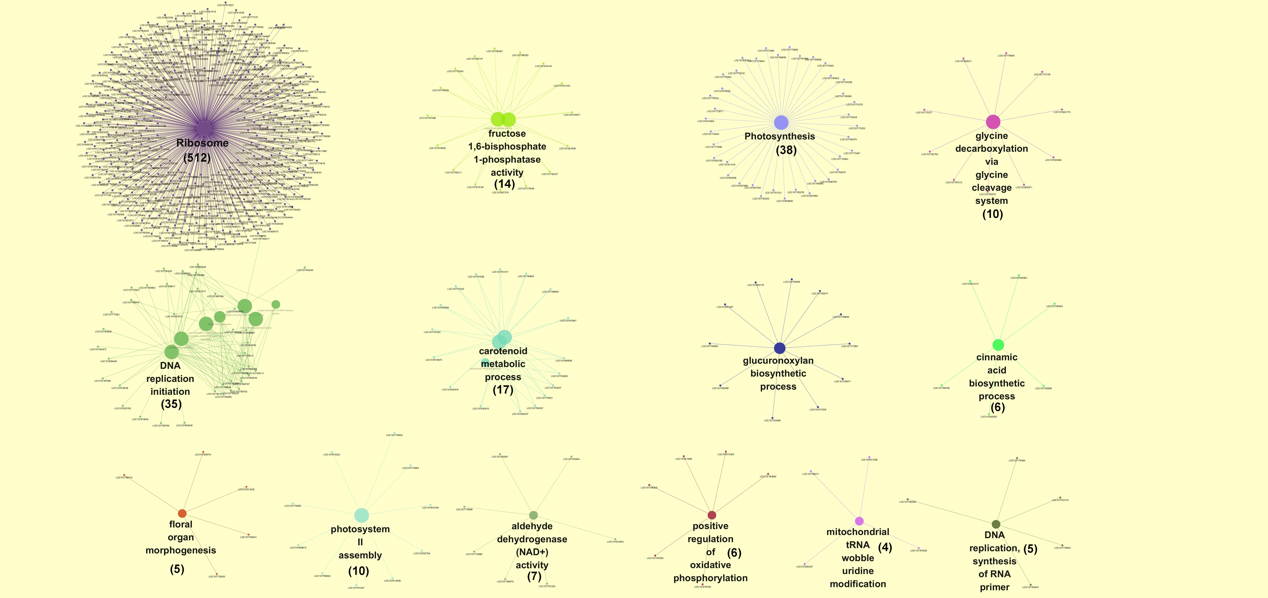


**Supplementary Figure 2.** Significant pathways or terms of differentially expressed genes (DEGs) annotated by ClueGO. (The circular pie indicates the corresponding pathway or term, and the number in the bracket indicates the number of genes in the corresponding pathway or term. The scattered straight lines connected by the pies show the genes involved in the pathways or terms.)


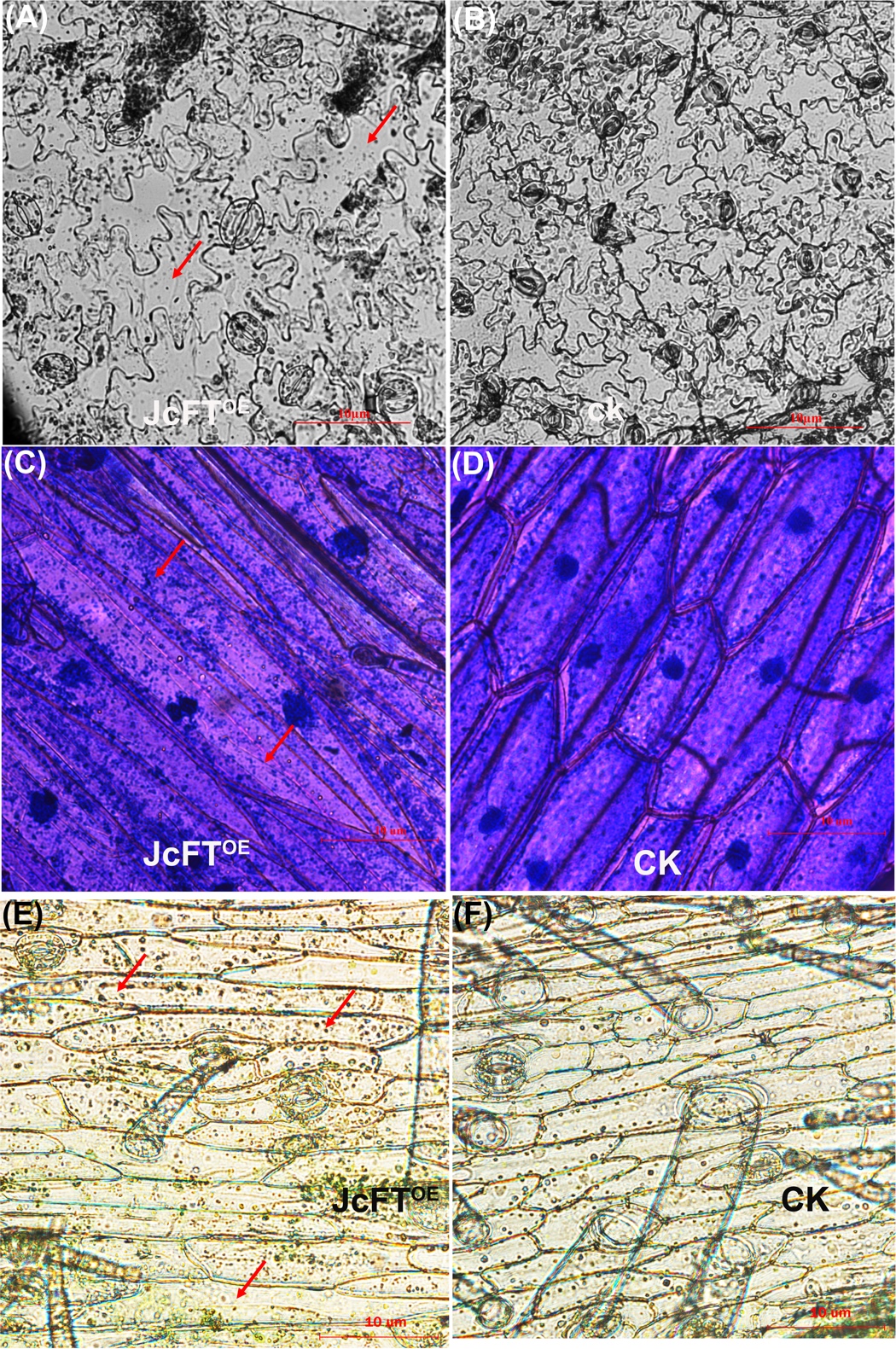


**Supplementary Figure 3.** Comparison of epidermal cells of leaves, petioles and stems of JcFT^OE^ and control. (The slices were prepared by the peeling method. (A, C, E) The sections of leaf, petiole and stem epidermal cells of JcFT^OE^, respectively. (B, D, F) The sections of leaf, petiole and stem epidermal cells of control, respectively. (C, D) The sections were prepared with reference to the previously described protocol (Waller and Nick 1997, Ahad and Nick 2007), and stained with Coomassie Brilliant Blue R250. Arrows indicate significantly longer cells in the JcFT^OE^ samples. scale bar = 10 μm)

**
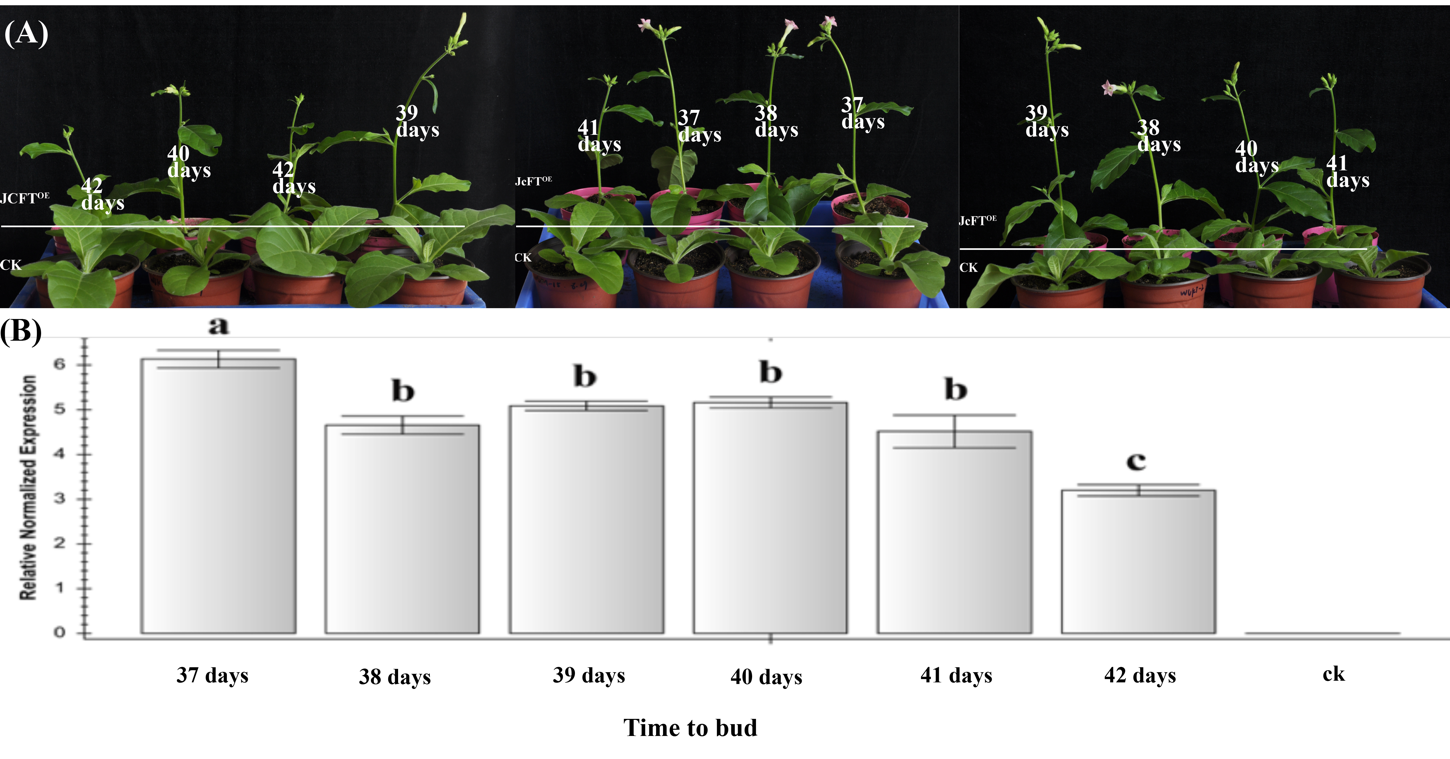
**

**Supplementary Figure 4.** Expression of JcFT in JcFT^OE^ plants at different flowering times. ((A) JcFT^OE^ plants flowering from 37 to 42 days after sowing, and CK. (B) Reverse-transcription analysis of JcFT expression in leaves of JcFT^OE^ plants at different flowering times. Two plant samples at each flowering stage were collected, mixed and ground, and RNA was extracted. Bars represented gene expression mean and standard error of mean, n=3, a, b, c showed significant differences.)

# Supplementary Tables

**Supplementary Table 1.** The primers for PCR and qRT-PCR

|  | **Accession number/Gene symbol** | **Forward Primer (FP)** | **Reverse Primer (RP)** | **Length of PCR product (bp)** | **FP TM (℃)** | **RP TM (℃)** | **FP GC%** | **RP GC%** |
| --- | --- | --- | --- | --- | --- | --- | --- | --- |
| *NPTII* | X57709 | GGAGAGGCTATTCGGCTATGAC | CACCATGATATCGGCAAGCAG | 547 | 59 | 58 | 55 | 50 |
| *JcFT* | NM_001308752 | CTAGCCCAAGTGACCCTAATCTC | GGGCTCTCATAGCACACTATCTC | 109 | 58 | 58 | 52 | 52 |
| *EF-1a* | AF120093 | TGAGATGCACCACGAAGCTC | CCAACATTGTCACCAGGAAGTG | 51 | 59 | 59 | 55 | 50 |
| *L25* | L18908 | CCCCTCACCACAGAGTCTGC | AAGGGTGTTGTTGTCCTCAATCTT | 51 | 62 | 59 | 65 | 42 |
| 14-3-3 protein related genes | LOC107818314 | TTCGGCTTACAAGTCCGCTC | AAAGGCCTGTTTGGCGAGAT | 151 | 60 | 59 | 50 | 55 |
|  | LOC107828339 | AATCCGACTTGGACTGGCTC | TTGCAAGCACGATCAGGAGA | 75 | 60 | 60 | 55 | 55 |
|  | LOC107784347 | GGCCAAGCAAGCATTTGACG | CTAGTTCTCCCCTTGACGCTC | 175 | 61 | 60 | 55 | 57 |
|  | LOC107794654 | CCGAACGCTACGAGGAGATG | CTCGATGGTGAGTTCGTCGT | 74 | 60 | 60 | 60 | 55 |
|  | LOC107801900 | TCGTGCCTGTAATCTTGCCA | AATGTGTCCAGCTCAGCGAT | 60 | 60 | 60 | 50 | 50 |
|  | LOC107805373 | TTAAATTGAGGCGCGTTGCG | TCGGTCCCCAGCATTCAAAG | 153 | 60 | 60 | 50 | 55 |
|  | LOC107811808 | CGAGCGTTACGAGGAGATGG | GTTCAGAGGAACCCGAGACG | 65 | 60 | 60 | 60 | 60 |
| DNA replication initiation related genes | LOC107818438 | AGAAGCTACGAACACCAGCC | TGGCGATTCAGATACCGACG | 98 | 60 | 60 | 55 | 55 |
|  | LOC107819504 | TGCCCACAATTAGCCGTGAT | GACGCATCGACGAGGAGATT | 161 | 60 | 60 | 50 | 55 |
|  | LOC107820754 | ATGCTGCTGATCAAGTCCGT | TTTGCGCCTTTTTGCCTGAG | 152 | 60 | 60 | 50 | 50 |
|  | LOC107821293 | CGGCGTATGCTTCAAAGTCC | AAAGCACGGGGAAAATGACG | 172 | 60 | 59 | 55 | 50 |
|  | LOC107760626 | AAGCCAACTACACGCGATCA | GCTTCACTTAACTTGCGGGC | 112 | 60 | 60 | 50 | 55 |
|  | LOC107770621 | CCCATCTCTCTCCAACTCGC | AGCGATTAACGGCAGAGGAG | 86 | 60 | 60 | 60 | 50 |
|  | LOC107771366 | AGTCCGCCCATCTCAATAGC | GACTGCGCTCGACATTCATC | 96 | 60 | 59 | 55 | 55 |
|  | LOC107780880 | GAGCTTCTGCCTCTAAGCCG | ACGGAGAACGTAGCCAAGTC | 98 | 61 | 60 | 60 | 55 |
|  | LOC107789612 | GCACTTGTAGCAGTTGCCTC | CGGGGATTGTCGGCATTTTG | 176 | 59 | 60 | 55 | 55 |
|  | LOC107809404 | CTTCTGTGCTCGGCTTTTGG | CACCGAATGCTCCGGGTAAT | 86 | 60 | 60 | 55 | 55 |
| IRX related genes | LOC107759843 | GTTGATTGCAGCAGCGAAGG | TGCTTCAGGCCAATACCCTC | 166 | 60 | 60 | 55 | 55 |
|  | LOC107760089 | CCCCACGATTTTGGTGCTTG | GAGGGATCAAGTGGGACTGC | 190 | 60 | 60 | 55 | 60 |
|  | LOC107762480 | CTCCAACGAGCGCGAAAGAT | TTGCTCCACAACCACCCATA | 104 | 61 | 59 | 55 | 50 |
|  | LOC107764680 | TCCAACGAGCGCGAAAGAT | TTTGTTGCTCCACAACCACC | 107 | 60 | 59 | 53 | 50 |
|  | LOC107765203 | GCGGCCTTGAGTCCTAGTTC | CACGAGCAATCTTCACCGTC | 161 | 60 | 59 | 60 | 55 |
|  | LOC107769571 | TCCCCTCCGGTGAAAAGTTG | CGGTCGTCCATGTTCTTTGC | 124 | 60 | 60 | 55 | 55 |
|  | LOC107770483 | GCTCCTGCTCCTACGCCTAT | AAAGGAAACAGCCGGTTCAG | 78 | 61 | 59 | 60 | 50 |
|  | LOC107778308 | CCCTCCACACTACACCTAGC | CCGGAGCCTGAGCAAAAATG | 121 | 59 | 60 | 60 | 55 |
|  | LOC107778315 | TTGGCATTTTGGCTCAAGGTC | AGCTGAAGCTCGAGTAGGTG | 62 | 60 | 59 | 48 | 55 |
|  | LOC107790336 | CGGCCTTACACATGAGACCC | TTCGCTGCTGCAATCAACTC | 180 | 60 | 59 | 60 | 50 |
|  | LOC107793555 | TTGCCTCGCTCCTTACTGTG | TGCAGAAACTACAGAGGCCG | 65 | 60 | 60 | 55 | 55 |
|  | LOC107793917 | TCAGGAAGTGCTGGTGATGTC | ACATGTGGTTGAGGCATCGT | 127 | 60 | 60 | 52 | 50 |
|  | LOC107796855 | TTGTGCTTCATGGGGTTTGC | GGAGCTGACGATGAGCTGTT | 106 | 60 | 60 | 50 | 55 |
|  | LOC107801367 | TCAACCAGGTGAGGCTTTCC | GCAGCTCGATCCTACCAAGG | 144 | 60 | 60 | 55 | 60 |
|  | LOC107803998 | CAGAGTGCAAACTCGGGCTA | AGCTGACATTCGACCAGGTG | 119 | 60 | 60 | 55 | 55 |
|  | LOC107809178 | GCTTGCTCGGAAATTGCCTC | GACCTGCTGTCCAGTTGAGT | 77 | 60 | 60 | 55 | 55 |
|  | LOC107812975 | ATGATTCCGGTGCTGAGAGC | ACGAGCAATCTTCACCGTCC | 114 | 60 | 60 | 55 | 55 |
|  | LOC107817639 | CCATGGACGACCTCTGCAAT | TGAAGCTGTAGAGGGCAAGC | 78 | 60 | 60 | 55 | 55 |
|  | LOC107817758 | TAGAGCCTCGAGCACCTGAT | GCGGTCCTTGACGAAGGTAG | 86 | 60 | 60 | 55 | 60 |
|  | LOC107820514 | TTCGTCAAGGACCGCAGAG | GGCCATGGAGTTCCGTCTT | 128 | 60 | 60 | 58 | 58 |
|  | LOC107828491 | TTCGATGGGCTCTTGGATCG | GGTGTAGGCAAGTCGTTGGA | 107 | 60 | 60 | 55 | 55 |
|  | LOC107830312 | TTGGGGGCCTTTATTTGGCA | GGTGTTCGATTTTGGCGTCC | 99 | 60 | 60 | 50 | 55 |
|  | LOC107790572 | TGTTTCAGAGAAGCGGCCAA | GACAGGTCCAGATGGCTTCC | 190 | 60 | 60 | 50 | 60 |
|  | LOC107769133 | GGTTTCAAGATGCACTGCCG | GGAACCAAGAGCCCATCGAA | 129 | 60 | 60 | 55 | 55 |
|  | LOC107806628 | ATTGCTGGAATCCGCTCACA | TGCTCTCCGTTTGCATGACT | 68 | 60 | 60 | 50 | 50 |

**Supplementary Table 2.** The evaluation of the sequencing output data of each sample

| Samples | Total Reads | Mapped Reads | Obtained Bases(bp) | Q20(%) | Q30(%) | GC(%) |
| --- | --- | --- | --- | --- | --- | --- |
| CK1 | 43,227,852 | 41015739(94.88%) | 6,435,264,102 | 96.86 | 91.83 | 43.74 |
| CK2 | 44,251,676 | 42121528(95.19%) | 6,603,766,328 | 97.22 | 92.61 | 43.53 |
| CK3 | 42,956,590 | 40719455(94.79%) | 6,402,646,436 | 96.8 | 91.76 | 43.56 |
| JcFT1 | 42,160,102 | 40054794(95.01%) | 6,285,912,696 | 97.05 | 92.21 | 43.62 |
| JcFT2 | 43,938,032 | 41736877(94.99%) | 6,552,791,746 | 97.05 | 92.23 | 43.7 |
| JcFT3 | 44,118,142 | 41827653(94.81%) | 6,581,964,718 | 96.99 | 92.05 | 43.82 |

**Supplementary Table 3.** Statistics of the expression level of 64 DEGs (differentially expressed genes) annotated as DNA replication in different samples

| Gene symbol | CK1_count | CK2_count | CK3_count | JcFT1_count | JcFT2_count | JcFT3_count | FDR^1^ | log2FC (JcFT / CK) | regulated |
| --- | --- | --- | --- | --- | --- | --- | --- | --- | --- |
| LOC107760626 | 252 | 276 | 140 | 13 | 37 | 108 | 0.002655 | -1.960791 | down |
| LOC107770621 | 272 | 298 | 121 | 9 | 38 | 84 | 0.003511 | -2.270583 | down |
| LOC107829689 | 229 | 224 | 186 | 38 | 81 | 125 | 0.00775 | -1.232112 | down |
| LOC107831517 | 213 | 142 | 156 | 55 | 46 | 59 | 0.002046 | -1.488478 | down |
| LOC107793883 | 161 | 131 | 143 | 36 | 31 | 64 | 0.002399 | -1.569301 | down |
| LOC107793571 | 250 | 275 | 196 | 19 | 30 | 71 | 1.03E-07 | -2.445618 | down |
| LOC107814297 | 198 | 234 | 176 | 50 | 28 | 86 | 0.00022 | -1.729396 | down |
| LOC107823792 | 748 | 753 | 507 | 117 | 124 | 263 | 3.77E-07 | -1.836944 | down |
| LOC107771390 | 341 | 326 | 198 | 50 | 66 | 121 | 0.000207 | -1.709337 | down |
| LOC107789473 | 457 | 423 | 335 | 70 | 47 | 157 | 2.59E-07 | -2.003537 | down |
| LOC107782073 | 160 | 137 | 149 | 28 | 27 | 62 | 0.000587 | -1.777601 | down |
| LOC107796644 | 757 | 858 | 489 | 33 | 55 | 218 | 3.92E-07 | -2.667823 | down |
| LOC107822467 | 864 | 1028 | 599 | 53 | 90 | 391 | 0.000145 | -2.112063 | down |
| LOC107830832 | 747 | 702 | 582 | 199 | 284 | 332 | 0.00086 | -1.136039 | down |
| LOC107792496 | 278 | 307 | 183 | 71 | 42 | 125 | 0.000433 | -1.530236 | down |
| LOC107828402 | 526 | 456 | 304 | 146 | 144 | 203 | 0.002035 | -1.202808 | down |
| LOC107779384 | 520 | 429 | 346 | 20 | 39 | 132 | 5.13E-09 | -2.648304 | down |
| LOC107784359 | 459 | 419 | 274 | 34 | 74 | 86 | 1.13E-07 | -2.398584 | down |
| LOC107775913 | 120 | 146 | 121 | 3 | 11 | 34 | 3.66E-07 | -2.897299 | down |
| LOC107809403 | 509 | 778 | 395 | 82 | 88 | 216 | 0.000458 | -1.967868 | down |
| LOC107809404 | 209 | 106 | 141 | 6 | 4 | 61 | 0.001223 | -2.616275 | down |
| LOC107818438 | 86 | 103 | 67 | 5 | 3 | 7 | 2.85E-09 | -3.918352 | down |
| LOC107820754 | 75 | 59 | 76 | 0 | 0 | 27 | 1.13E-05 | -2.921474 | down |
| LOC107775468 | 341 | 302 | 200 | 79 | 101 | 100 | 0.000697 | -1.397936 | down |
| LOC107760761 | 334 | 409 | 260 | 111 | 114 | 156 | 0.002221 | -1.209983 | down |
| LOC107819977 | 155 | 220 | 149 | 40 | 54 | 93 | 0.006836 | -1.319006 | down |
| LOC107776893 | 845 | 738 | 584 | 315 | 214 | 431 | 0.003564 | -1.001337 | down |
| LOC107784237 | 2384 | 2342 | 1300 | 143 | 250 | 796 | 0.000195 | -2.220099 | down |
| LOC107805042 | 2086 | 1971 | 1154 | 135 | 192 | 595 | 2.31E-05 | -2.371967 | down |
| LOC107783151 | 2564 | 2539 | 1453 | 114 | 183 | 539 | 2.41E-07 | -2.843799 | down |
| LOC107799111 | 1792 | 1850 | 1091 | 63 | 92 | 497 | 4.85E-07 | -2.759335 | down |
| LOC107804766 | 1508 | 1610 | 982 | 80 | 86 | 357 | 1.50E-09 | -2.850175 | down |
| LOC107805416 | 2291 | 2277 | 1489 | 172 | 157 | 689 | 1.02E-07 | -2.451824 | down |
| LOC107774668 | 1951 | 1948 | 1222 | 145 | 171 | 593 | 8.06E-07 | -2.367672 | down |
| LOC107795484 | 2487 | 2155 | 1562 | 213 | 320 | 720 | 6.92E-07 | -2.166231 | down |
| LOC107795499 | 1769 | 1825 | 1100 | 92 | 118 | 574 | 3.44E-06 | -2.474033 | down |
| LOC107799705 | 2761 | 2760 | 1659 | 144 | 209 | 770 | 9.61E-07 | -2.558479 | down |
| LOC107805831 | 3000 | 2984 | 1851 | 230 | 280 | 919 | 1.73E-06 | -2.326468 | down |
| LOC107808167 | 3228 | 3218 | 1935 | 222 | 274 | 942 | 2.36E-06 | -2.417391 | down |
| LOC107814793 | 785 | 731 | 398 | 44 | 125 | 280 | 0.00194 | -1.961657 | down |
| LOC107820400 | 609 | 656 | 444 | 189 | 223 | 311 | 0.002683 | -1.061954 | down |
| LOC107791387 | 1332 | 1198 | 1016 | 84 | 89 | 320 | 1.04E-16 | -2.720997 | down |
| LOC107805236 | 1000 | 1072 | 676 | 81 | 74 | 276 | 4.56E-09 | -2.54163 | down |
| LOC107789816 | 391 | 402 | 304 | 124 | 103 | 173 | 0.001002 | -1.276508 | down |
| LOC107777381 | 136 | 137 | 112 | 15 | 0 | 21 | 1.39E-08 | -3.270466 | down |
| LOC107784473 | 120 | 136 | 65 | 17 | 26 | 18 | 0.000129 | -2.185927 | down |
| LOC107787095 | 135 | 121 | 110 | 27 | 37 | 58 | 0.008921 | -1.419804 | down |
| LOC107788817 | 138 | 110 | 96 | 16 | 32 | 38 | 0.000899 | -1.83026 | down |
| LOC107795768 | 189 | 131 | 68 | 8 | 10 | 25 | 0.001509 | -3.037081 | down |
| LOC107800430 | 132 | 149 | 64 | 3 | 10 | 25 | 5.71E-05 | -3.056993 | down |
| LOC107809180 | 319 | 317 | 191 | 0 | 10 | 56 | 3.33E-10 | -3.56997 | down |
| LOC107778290 | 2216 | 2222 | 1686 | 401 | 324 | 781 | 2.91E-09 | -1.865026 | down |
| LOC107779602 | 2091 | 2066 | 1482 | 132 | 192 | 523 | 6.11E-13 | -2.601178 | down |
| LOC107823681 | 1320 | 1152 | 801 | 47 | 88 | 419 | 7.39E-06 | -2.46404 | down |
| LOC107790306 | 1262 | 1215 | 731 | 83 | 137 | 402 | 2.76E-05 | -2.240186 | down |
| LOC107780880 | 159 | 172 | 113 | 13 | 22 | 32 | 1.32E-06 | -2.561109 | down |
| LOC107821293 | 238 | 204 | 156 | 18 | 24 | 45 | 7.49E-08 | -2.626067 | down |
| LOC107785850 | 1295 | 1268 | 741 | 47 | 105 | 330 | 1.77E-06 | -2.659866 | down |
| LOC107796715 | 282 | 284 | 179 | 47 | 52 | 113 | 0.000167 | -1.658519 | down |
| LOC107765327 | 368 | 438 | 295 | 100 | 79 | 261 | 0.009081 | -1.179137 | down |
| LOC107813256 | 170 | 192 | 139 | 39 | 43 | 87 | 0.004603 | -1.407222 | down |
| LOC107763347 | 76 | 92 | 75 | 11 | 23 | 27 | 0.002273 | -1.8182 | down |
| LOC107771366 | 546 | 523 | 356 | 52 | 62 | 120 | 2.90E-10 | -2.44816 | down |
| LOC107820701 | 635 | 643 | 414 | 51 | 52 | 145 | 5.19E-10 | -2.627413 | down |

FDR: false discovery rate; FC: fold change

**Supplementary Table 4.** DEGs (differentially expressed genes) related to cell cycle regulation annotated by ClueGO

| Gene symbol | CK1_count | CK2_count | CK3_count | JcFT1_count | JcFT2_count | JcFT3_count | FDR | log2FC (JcFT / CK) | regulated |
| --- | --- | --- | --- | --- | --- | --- | --- | --- | --- |
| LOC107760504 | 283 | 280 | 210 | 8 | 84 | 147 | 0.001792 | -1.562284 | down |
| LOC107760626 | 252 | 276 | 140 | 13 | 37 | 108 | 0.002655 | -1.960791 | down |
| LOC107762855 | 1885 | 1987 | 1832 | 556 | 559 | 847 | 1.54E-05 | -1.361014 | down |
| LOC107763347 | 76 | 92 | 75 | 11 | 23 | 27 | 0.002273 | -1.8182 | down |
| LOC107766317 | 226 | 199 | 213 | 599 | 610 | 349 | 0.004991 | 1.510679 | up |
| LOC107766674 | 85 | 99 | 118 | 17 | 5 | 62 | 0.003323 | -1.736517 | down |
| LOC107770621 | 272 | 298 | 121 | 9 | 38 | 84 | 0.003511 | -2.270583 | down |
| LOC107771366 | 546 | 523 | 356 | 52 | 62 | 120 | 2.90E-10 | -2.44816 | down |
| LOC107771927 | 514 | 466 | 401 | 904 | 884 | 830 | 0.000525 | 1.12531 | up |
| LOC107772402 | 665 | 838 | 1038 | 176 | 182 | 390 | 1.15E-05 | -1.604576 | down |
| LOC107773243 | 77 | 65 | 56 | 5 | 0 | 38 | 0.001118 | -2.1336 | down |
| LOC107775913 | 120 | 146 | 121 | 3 | 11 | 34 | 3.66E-07 | -2.897299 | down |
| LOC107776537 | 140 | 129 | 156 | 9 | 16 | 49 | 9.23E-06 | -2.400377 | down |
| LOC107779596 | 18 | 51 | 43 | 143 | 135 | 122 | 5.43E-05 | 2.056267 | up |
| LOC107780223 | 2505 | 2566 | 2213 | 361 | 775 | 736 | 1.20E-08 | -1.775811 | down |
| LOC107780802 | 271 | 177 | 104 | 3 | 3 | 40 | 0.000505 | -3.51801 | down |
| LOC107780880 | 159 | 172 | 113 | 13 | 22 | 32 | 1.32E-06 | -2.561109 | down |
| LOC107782018 | 599 | 586 | 436 | 60 | 82 | 378 | 0.008831 | -1.532359 | down |
| LOC107782163 | 1710 | 1891 | 1785 | 513 | 569 | 1027 | 0.000229 | -1.186391 | down |
| LOC107783502 | 375 | 378 | 315 | 1214 | 1207 | 869 | 3.38E-06 | 1.839172 | up |
| LOC107787921 | 825 | 806 | 700 | 3 | 35 | 233 | 1.99E-17 | -3.030105 | down |
| LOC107788314 | 1002 | 1159 | 1125 | 3319 | 2836 | 1867 | 0.004192 | 1.513113 | up |
| LOC107789612 | 340 | 225 | 170 | 0 | 12 | 49 | 7.52E-06 | -3.509904 | down |
| LOC107791668 | 72 | 72 | 60 | 7 | 10 | 37 | 0.004744 | -1.799531 | down |
| LOC107795100 | 212 | 256 | 134 | 32 | 27 | 85 | 0.000332 | -1.918442 | down |
| LOC107797635 | 100 | 88 | 62 | 18 | 14 | 38 | 0.005018 | -1.685403 | down |
| LOC107797636 | 95 | 89 | 57 | 4 | 24 | 40 | 0.005712 | -1.68839 | down |
| LOC107798635 | 497 | 596 | 460 | 51 | 44 | 180 | 2.14E-10 | -2.368353 | down |
| LOC107804529 | 302 | 199 | 144 | 7 | 21 | 96 | 0.005367 | -2.289917 | down |
| LOC107805580 | 336 | 335 | 218 | 85 | 100 | 149 | 0.002716 | -1.23805 | down |
| LOC107809288 | 324 | 392 | 295 | 52 | 51 | 137 | 2.25E-06 | -1.92584 | down |
| LOC107809403 | 509 | 778 | 395 | 82 | 88 | 216 | 0.000458 | -1.967868 | down |
| LOC107809404 | 209 | 106 | 141 | 6 | 4 | 61 | 0.001223 | -2.616275 | down |
| LOC107811824 | 163 | 182 | 177 | 22 | 30 | 115 | 0.002532 | -1.525495 | down |
| LOC107812739 | 595 | 500 | 390 | 34 | 33 | 156 | 1.26E-08 | -2.622697 | down |
| LOC107813910 | 2751 | 2709 | 2942 | 819 | 1030 | 1838 | 0.002293 | -1.026895 | down |
| LOC107817396 | 133 | 141 | 124 | 0 | 13 | 24 | 9.83E-09 | -3.303511 | down |
| LOC107817613 | 580 | 474 | 497 | 236 | 203 | 228 | 0.003952 | -1.021586 | down |
| LOC107818438 | 86 | 103 | 67 | 5 | 3 | 7 | 2.85E-09 | -3.918352 | down |
| LOC107818682 | 255 | 245 | 261 | 833 | 912 | 841 | 8.28E-10 | 1.965723 | up |
| LOC107819504 | 127 | 114 | 80 | 20 | 15 | 49 | 0.001674 | -1.792115 | down |
| LOC107820701 | 635 | 643 | 414 | 51 | 52 | 145 | 5.19E-10 | -2.627413 | down |
| LOC107820754 | 75 | 59 | 76 | 0 | 0 | 27 | 1.13E-05 | -2.921474 | down |
| LOC107821036 | 163 | 152 | 126 | 5 | 9 | 52 | 1.30E-06 | -2.648102 | down |
| LOC107821293 | 238 | 204 | 156 | 18 | 24 | 45 | 7.49E-08 | -2.626067 | down |
| LOC107822351 | 219 | 169 | 117 | 20 | 26 | 89 | 0.005461 | -1.783129 | down |
| LOC107822860 | 32 | 40 | 27 | 75 | 108 | 96 | 0.00217 | 1.694916 | up |
| LOC107824914 | 180 | 152 | 113 | 43 | 49 | 50 | 0.003747 | -1.454975 | down |
| LOC107828050 | 136 | 115 | 63 | 17 | 13 | 42 | 0.002958 | -1.983703 | down |
| LOC107829247 | 1347 | 1393 | 1501 | 335 | 342 | 752 | 1.04E-05 | -1.413086 | down |
| LOC107829689 | 229 | 224 | 186 | 38 | 81 | 125 | 0.00775 | -1.232112 | down |
| LOC107830644 | 259 | 274 | 187 | 62 | 50 | 113 | 0.000615 | -1.514563 | down |
| LOC107831050 | 587 | 616 | 505 | 197 | 207 | 299 | 0.001797 | -1.100698 | down |
| LOC107831211 | 105 | 98 | 76 | 5 | 5 | 16 | 1.41E-07 | -3.28941 | down |
| LOC107832697 | 722 | 950 | 1369 | 205 | 217 | 472 | 0.002503 | -1.60797 | down |

FDR: false discovery rate; FC: fold change

**Supplementary Table 5.** The DEGs (differentially expressed genes) annotated by BiNGO as sucrose metabolism

| Gene symbol | FDR | log2FC (JcFT / CK) | Regulated | KEGG_annotation | Pfam_annotation | Swiss_Prot_annotation |
| --- | --- | --- | --- | --- | --- | --- |
| LOC107760211 | 0.000692 | 1.176495 | up | K03841\|0\|spen:107027028\|K03841 fructose-1,6-bisphosphatase I [EC:3.1.3.11] \| (RefSeq) fructose-1,6-bisphosphatase class 1 | Fructose-1-6-bisphosphatase, N-terminal domain | Fructose-1,6-bisphosphatase, chloroplastic OS=Brassica napus OX=3708 GN=FBP PE=2 SV=1 |
| LOC107775549 | 1.40E-05 | 2.587922 | up | K01100\|0\|spen:107020395\|K01100 sedoheptulose-bisphosphatase [EC:3.1.3.37] \| (RefSeq) sedoheptulose-1,7-bisphosphatase, chloroplastic | Fructose-1-6-bisphosphatase, N-terminal domain | Sedoheptulose-1,7-bisphosphatase, chloroplastic OS=Spinacia oleracea OX=3562 PE=2 SV=1 |
| LOC107779243 | 8.09E-13 | 4.196135 | up | K01100\|0\|sot:102583431\|K01100 sedoheptulose-bisphosphatase [EC:3.1.3.37] \| (RefSeq) sedoheptulose-1,7-bisphosphatase, chloroplastic | Fructose-1-6-bisphosphatase, N-terminal domain | Sedoheptulose-1,7-bisphosphatase, chloroplastic OS=Spinacia oleracea OX=3562 PE=2 SV=1 |
| LOC107784539 | 3.79E-05 | 4.474135 | up | K03841\|0\|sot:102590778\|K03841 fructose-1,6-bisphosphatase I [EC:3.1.3.11] \| (RefSeq) fructose-1,6-bisphosphatase, chloroplastic-like | Fructose-1-6-bisphosphatase, N-terminal domain | Fructose-1,6-bisphosphatase, chloroplastic OS=Brassica napus OX=3708 GN=FBP PE=2 SV=1 |
| LOC107784747 | 2.59E-07 | 3.398391 | up | K03841\|0\|sly:101260852\|K03841 fructose-1,6-bisphosphatase I [EC:3.1.3.11] \| (RefSeq) fructose-1,6-bisphosphatase, chloroplastic | Fructose-1-6-bisphosphatase, N-terminal domain | Fructose-1,6-bisphosphatase, chloroplastic OS=Pisum sativum OX=3888 GN=FBP PE=1 SV=2 |
| LOC107788283 | 2.68E-17 | 6.376105 | up | K03841\|0\|sly:101266629\|K03841 fructose-1,6-bisphosphatase I [EC:3.1.3.11] \| (RefSeq) fructose-1,6-bisphosphatase, cytosolic | Fructose-1-6-bisphosphatase, N-terminal domain | Fructose-1,6-bisphosphatase, cytosolic OS=Solanum tuberosum OX=4113 PE=2 SV=1 |
| LOC107792237 | 6.11E-08 | 3.350859 | up | K03841\|0\|sot:102577434\|K03841 fructose-1,6-bisphosphatase I [EC:3.1.3.11] \| (RefSeq) fructose-1,6-bisphosphatase precursor | Fructose-1-6-bisphosphatase, N-terminal domain | Fructose-1,6-bisphosphatase, chloroplastic OS=Pisum sativum OX=3888 GN=FBP PE=1 SV=2 |
| LOC107807379 | 1.70E-07 | 6.018023 | up | K03841\|0\|sly:101266629\|K03841 fructose-1,6-bisphosphatase I [EC:3.1.3.11] \| (RefSeq) fructose-1,6-bisphosphatase, cytosolic | Fructose-1-6-bisphosphatase, N-terminal domain | Fructose-1,6-bisphosphatase, cytosolic OS=Solanum tuberosum OX=4113 PE=2 SV=1 |
| LOC107816146 | 9.58E-19 | 7.211096 | up | K01100\|0\|sot:102583431\|K01100 sedoheptulose-bisphosphatase [EC:3.1.3.37] \| (RefSeq) sedoheptulose-1,7-bisphosphatase, chloroplastic | Fructose-1-6-bisphosphatase, N-terminal domain | Sedoheptulose-1,7-bisphosphatase, chloroplastic OS=Spinacia oleracea OX=3562 PE=2 SV=1 |
| LOC107819136 | 0.001821 | 1.341946 | up | K03841\|0\|sot:102599458\|K03841 fructose-1,6-bisphosphatase I [EC:3.1.3.11] \| (RefSeq) fructose-1,6-bisphosphatase, cytosolic-like | Fructose-1-6-bisphosphatase, N-terminal domain | Fructose-1,6-bisphosphatase, cytosolic OS=Solanum tuberosum OX=4113 PE=2 SV=1 |
| LOC107819549 | 5.93E-05 | 1.69263 | up | K03841\|0\|spen:107002346\|K03841 fructose-1,6-bisphosphatase I [EC:3.1.3.11] \| (RefSeq) fructose-1,6-bisphosphatase, chloroplastic-like | Fructose-1-6-bisphosphatase, N-terminal domain | Fructose-1,6-bisphosphatase, chloroplastic OS=Oryza sativa subsp. japonica OX=39947 GN=Os03g0267300 PE=2 SV=1 |
| LOC107819677 | 0.000762 | 2.046891 | up | K01100\|0\|spen:107020395\|K01100 sedoheptulose-bisphosphatase [EC:3.1.3.37] \| (RefSeq) sedoheptulose-1,7-bisphosphatase, chloroplastic | Fructose-1-6-bisphosphatase, N-terminal domain | Sedoheptulose-1,7-bisphosphatase, chloroplastic OS=Spinacia oleracea OX=3562 PE=2 SV=1 |
| LOC107827939 | 0.000852 | 1.294448 | up | K03841\|0\|spen:107027028\|K03841 fructose-1,6-bisphosphatase I [EC:3.1.3.11] \| (RefSeq) fructose-1,6-bisphosphatase class 1 | Fructose-1-6-bisphosphatase, N-terminal domain | Fructose-1,6-bisphosphatase, chloroplastic OS=Brassica napus OX=3708 GN=FBP PE=2 SV=1 |
| LOC107831320 | 0.000487 | 2.294509 | up | K03841\|0\|sot:102599458\|K03841 fructose-1,6-bisphosphatase I [EC:3.1.3.11] \| (RefSeq) fructose-1,6-bisphosphatase, cytosolic-like | Fructose-1-6-bisphosphatase, N-terminal domain | Fructose-1,6-bisphosphatase, cytosolic OS=Solanum tuberosum OX=4113 PE=2 SV=1 |

FDR: false discovery rate; FC: fold change

**Supplementary Table 6.** DEGs (differentially expressed genes) closely related to the cytoskeleton annotated by KOG (clusters of orthologous groups for eukaryotic complete genomes) classification

| Gene symbol | CK1_count | CK2_count | CK3_count | JcFT1_count | JcFT2_count | JcFT3_count | FDR | log2FC (JcFT / CK) | Regulated | KOG_class_annotation |
| --- | --- | --- | --- | --- | --- | --- | --- | --- | --- | --- |
| LOC107832584 | 661 | 677 | 810 | 113 | 290 | 253 | 4.94E-06 | -1.526168 | down | Cytoskeleton |
| LOC107828576 | 696 | 754 | 552 | 95 | 173 | 460 | 0.004874 | -1.328044 | down | Cytoskeleton |
| LOC107826670 | 300 | 356 | 329 | 87 | 109 | 179 | 0.002424 | -1.224125 | down | Cytoskeleton |
| LOC107826020 | 96 | 149 | 89 | 44 | 23 | 36 | 0.005867 | -1.490758 | down | Cytoskeleton |
| LOC107821956 | 2832 | 2820 | 2222 | 611 | 883 | 1184 | 1.54E-05 | -1.381643 | down | Cytoskeleton |
| LOC107821481 | 12387 | 12117 | 9222 | 3308 | 4724 | 4682 | 0.001456 | -1.216658 | down | Cytoskeleton |
| LOC107820655 | 721 | 670 | 513 | 149 | 195 | 409 | 0.001756 | -1.187949 | down | Cytoskeleton |
| LOC107816247 | 352 | 426 | 199 | 107 | 84 | 143 | 0.007668 | -1.365043 | down | Cytoskeleton |
| LOC107815305 | 93 | 136 | 95 | 11 | 18 | 24 | 1.90E-05 | -2.434515 | down | Cytoskeleton |
| LOC107810980 | 236 | 248 | 168 | 35 | 74 | 143 | 0.008396 | -1.225895 | down | Cytoskeleton |
| LOC107809641 | 185 | 158 | 121 | 26 | 26 | 68 | 0.000437 | -1.806798 | down | Cytoskeleton |
| LOC107807750 | 428 | 443 | 326 | 95 | 116 | 199 | 0.000315 | -1.380078 | down | Cytoskeleton |
| LOC107807261 | 169 | 207 | 169 | 22 | 46 | 130 | 0.00743 | -1.333744 | down | Cytoskeleton |
| LOC107805059 | 242 | 258 | 197 | 50 | 90 | 106 | 0.002717 | -1.325303 | down | Cytoskeleton |
| LOC107796649 | 3245 | 3380 | 1907 | 348 | 610 | 622 | 1.50E-06 | -2.247339 | down | Cytoskeleton |
| LOC107793808 | 985 | 1015 | 1394 | 320 | 430 | 563 | 0.000214 | -1.19267 | down | Cytoskeleton |
| LOC107793296 | 202 | 262 | 180 | 5 | 31 | 49 | 8.94E-09 | -2.776788 | down | Cytoskeleton |
| LOC107789756 | 175 | 163 | 97 | 25 | 35 | 77 | 0.004243 | -1.520863 | down | Cytoskeleton |
| LOC107787874 | 312 | 359 | 263 | 78 | 70 | 208 | 0.002894 | -1.246333 | down | Cytoskeleton |
| LOC107775153 | 5793 | 6377 | 5527 | 1185 | 2208 | 3595 | 0.000974 | -1.182124 | down | Cytoskeleton |
| LOC107763690 | 985 | 1570 | 1698 | 236 | 254 | 843 | 0.003783 | -1.537032 | down | Cytoskeleton |
| LOC107759643 | 107 | 58 | 89 | 4 | 6 | 6 | 9.67E-09 | -3.808195 | down | Cytoskeleton |
| newGene_12312 | 197 | 175 | 147 | 18 | 20 | 120 | 0.007639 | -1.616926 | down | Cytoskeleton |
| newGene_33123 | 453 | 446 | 453 | 107 | 144 | 267 | 0.001117 | -1.226728 | down | Cytoskeleton |
| newGene_13644 | 720 | 776 | 680 | 253 | 277 | 276 | 0.000191 | -1.233853 | down | Cytoskeleton |
| LOC107832556 | 47 | 61 | 80 | 154 | 174 | 108 | 0.003537 | 1.435268 | up | Cytoskeleton |
| LOC107830354 | 684 | 767 | 833 | 1510 | 1989 | 1430 | 0.000104 | 1.319876 | up | Cytoskeleton |
| LOC107826880 | 5777 | 6274 | 8323 | 16025 | 22149 | 18584 | 5.76E-05 | 1.678983 | up | Cytoskeleton |
| LOC107826862 | 238 | 173 | 193 | 1107 | 1317 | 944 | 2.01E-11 | 2.685827 | up | Cytoskeleton |
| LOC107823471 | 2078 | 2460 | 3428 | 8335 | 12544 | 9232 | 5.33E-07 | 2.1245 | up | Cytoskeleton |
| LOC107822688 | 113 | 160 | 237 | 2192 | 2745 | 1859 | 5.20E-15 | 3.952203 | up | Cytoskeleton |
| LOC107820284 | 103 | 179 | 133 | 296 | 284 | 283 | 0.001498 | 1.266058 | up | Cytoskeleton |
| LOC107819171 | 299 | 247 | 238 | 518 | 601 | 514 | 0.000234 | 1.259478 | up | Cytoskeleton |
| LOC107818875 | 754 | 863 | 1074 | 6946 | 6356 | 3704 | 1.87E-05 | 2.887724 | up | Cytoskeleton |
| LOC107817925 | 208 | 228 | 239 | 1044 | 1114 | 739 | 4.94E-07 | 2.320217 | up | Cytoskeleton |
| LOC107816149 | 1361 | 1446 | 1404 | 2797 | 2779 | 3657 | 3.90E-05 | 1.319496 | up | Cytoskeleton |
| LOC107815935 | 218 | 186 | 301 | 794 | 715 | 465 | 0.001331 | 1.705483 | up | Cytoskeleton |
| LOC107813623 | 396 | 383 | 516 | 1253 | 1188 | 715 | 0.005079 | 1.508673 | up | Cytoskeleton |
| LOC107811983 | 30 | 27 | 19 | 114 | 109 | 127 | 6.44E-06 | 2.395559 | up | Cytoskeleton |
| LOC107811917 | 43 | 39 | 25 | 1276 | 1417 | 819 | 2.49E-13 | 5.257461 | up | Cytoskeleton |
| LOC107809316 | 0 | 9 | 47 | 965 | 764 | 235 | 0.00018 | 5.385848 | up | Cytoskeleton |
| LOC107802308 | 103 | 138 | 206 | 1208 | 995 | 438 | 0.00343 | 2.804052 | up | Cytoskeleton |
| LOC107801848 | 149 | 113 | 53 | 231 | 234 | 193 | 0.003113 | 1.267484 | up | Cytoskeleton |
| LOC107797817 | 84 | 119 | 77 | 512 | 449 | 285 | 8.64E-05 | 2.383832 | up | Cytoskeleton |
| LOC107797065 | 7120 | 8013 | 8905 | 16951 | 24790 | 14555 | 0.004603 | 1.444414 | up | Cytoskeleton |
| LOC107796502 | 49 | 52 | 110 | 311 | 293 | 139 | 0.008575 | 2.048934 | up | Cytoskeleton |
| LOC107792234 | 212 | 180 | 279 | 567 | 370 | 415 | 0.004015 | 1.216082 | up | Cytoskeleton |
| LOC107792118 | 99 | 102 | 140 | 1407 | 1228 | 402 | 0.003467 | 3.400311 | up | Cytoskeleton |
| LOC107791272 | 57 | 55 | 19 | 253 | 261 | 114 | 0.002623 | 2.495206 | up | Cytoskeleton |
| LOC107787766 | 569 | 531 | 598 | 3908 | 4191 | 2379 | 4.15E-06 | 2.847955 | up | Cytoskeleton |
| LOC107785893 | 131 | 142 | 438 | 1134 | 1149 | 700 | 0.000159 | 2.290395 | up | Cytoskeleton |
| LOC107782123 | 607 | 725 | 696 | 1064 | 1550 | 1100 | 0.002918 | 1.082364 | up | Cytoskeleton |
| LOC107779013 | 932 | 970 | 1315 | 8687 | 8325 | 5108 | 9.27E-07 | 3.005 | up | Cytoskeleton |
| LOC107777817 | 634 | 862 | 820 | 3360 | 3986 | 2497 | 2.46E-06 | 2.308993 | up | Cytoskeleton |
| LOC107776651 | 152 | 183 | 309 | 831 | 675 | 474 | 0.001277 | 1.843981 | up | Cytoskeleton |
| LOC107774378 | 273 | 241 | 264 | 787 | 638 | 653 | 8.31E-07 | 1.62082 | up | Cytoskeleton |
| LOC107774245 | 808 | 962 | 1412 | 2321 | 2287 | 1694 | 0.001987 | 1.202223 | up | Cytoskeleton |
| LOC107773558 | 4794 | 5493 | 5180 | 11807 | 11545 | 7784 | 0.003041 | 1.230324 | up | Cytoskeleton |
| LOC107771520 | 759 | 607 | 822 | 1548 | 1637 | 1099 | 0.003151 | 1.183156 | up | Cytoskeleton |
| LOC107769836 | 80 | 139 | 643 | 1099 | 1805 | 1164 | 0.000229 | 2.448333 | up | Cytoskeleton |
| LOC107768771 | 33 | 46 | 238 | 2749 | 3362 | 1508 | 1.54E-07 | 4.816339 | up | Cytoskeleton |
| LOC107767007 | 28 | 25 | 20 | 99 | 108 | 64 | 0.00019 | 2.11225 | up | Cytoskeleton |
| LOC107763431 | 1 | 2 | 31 | 2209 | 3132 | 1178 | 4.17E-10 | 7.814064 | up | Cytoskeleton |
| LOC107761206 | 23 | 17 | 7 | 235 | 158 | 156 | 9.63E-10 | 3.754144 | up | Cytoskeleton |
| LOC107760364 | 406 | 405 | 333 | 783 | 760 | 626 | 0.000668 | 1.133427 | up | Cytoskeleton |
| newGene_5645 | 364 | 330 | 410 | 858 | 816 | 602 | 0.000691 | 1.258021 | up | Cytoskeleton |
| newGene_20442 | 110 | 156 | 122 | 342 | 410 | 380 | 2.48E-06 | 1.749016 | up | Cytoskeleton |
| newGene_9445 | 84 | 103 | 205 | 483 | 676 | 383 | 0.000298 | 2.194817 | up | Cytoskeleton |
| newGene_19058 | 955 | 1042 | 871 | 3241 | 3098 | 2085 | 0.00012 | 1.775533 | up | Cytoskeleton |
| newGene_32933 | 7674 | 8186 | 8798 | 25778 | 32989 | 17048 | 0.002213 | 1.84449 | up | Cytoskeleton |

FDR: false discovery rate; FC: fold change

**Supplementary Table 7.** DEGs (differentially expressed genes) related to hemicellulose metabolism annotated by ClueGO

| Gene symbol | CK1_count | CK2_count | CK3_count | JcFT1_count | JcFT2_count | JcFT3_count | FDR | log2FC (JcFT / CK) | Regulated |
| --- | --- | --- | --- | --- | --- | --- | --- | --- | --- |
| LOC107759843 | 8 | 30 | 178 | 5894 | 4476 | 2739 | 2.71E-10 | 6.154913 | up |
| LOC107760089 | 648 | 746 | 1570 | 2420 | 5394 | 3864 | 0.001916 | 2.176294 | up |
| LOC107762480 | 94 | 104 | 383 | 2295 | 3464 | 2153 | 5.98E-10 | 3.978771 | up |
| LOC107764680 | 42 | 104 | 596 | 5398 | 6574 | 3633 | 1.80E-10 | 4.617732 | up |
| LOC107764751 | 406 | 424 | 3911 | 10531 | 17279 | 10947 | 1.54E-06 | 3.238532 | up |
| LOC107769571 | 777 | 958 | 1519 | 2876 | 4970 | 3676 | 5.12E-05 | 2.027097 | up |
| LOC107771244 | 78 | 86 | 620 | 2064 | 4536 | 2839 | 1.61E-05 | 3.792296 | up |
| LOC107772801 | 1506 | 1697 | 2357 | 3479 | 5486 | 3438 | 0.006717 | 1.369415 | up |
| LOC107773385 | 1784 | 1784 | 1846 | 8643 | 9965 | 5596 | 4.08E-05 | 2.382772 | up |
| LOC107773919 | 2128 | 2077 | 3856 | 5794 | 12732 | 8721 | 0.004328 | 1.956101 | up |
| LOC107775958 | 21 | 51 | 404 | 6272 | 6141 | 3393 | 7.90E-11 | 5.280567 | up |
| LOC107776309 | 979 | 834 | 715 | 4527 | 5481 | 4039 | 1.02E-12 | 2.681468 | up |
| LOC107776310 | 148 | 127 | 114 | 329 | 458 | 245 | 0.004947 | 1.625467 | up |
| LOC107778390 | 29 | 38 | 292 | 1732 | 2204 | 1287 | 6.94E-10 | 4.080241 | up |
| LOC107790336 | 13 | 40 | 223 | 5003 | 3770 | 2673 | 1.03E-11 | 5.600007 | up |
| LOC107791241 | 410 | 392 | 510 | 85 | 131 | 282 | 0.001134 | -1.252955 | down |
| LOC107791565 | 2512 | 2132 | 1541 | 9237 | 11315 | 6783 | 8.63E-06 | 2.359848 | up |
| LOC107792199 | 43 | 32 | 339 | 5640 | 5033 | 2477 | 8.16E-08 | 5.218894 | up |
| LOC107793555 | 5 | 45 | 309 | 3240 | 3738 | 1877 | 4.30E-09 | 4.853559 | up |
| LOC107793608 | 222 | 218 | 756 | 7594 | 8371 | 3551 | 6.19E-06 | 4.260242 | up |
| LOC107793917 | 579 | 632 | 937 | 1965 | 3590 | 2275 | 0.000724 | 2.073855 | up |
| LOC107796855 | 1339 | 1444 | 2139 | 4599 | 8102 | 5374 | 0.000229 | 2.083024 | up |
| LOC107800600 | 13 | 14 | 145 | 1394 | 1608 | 997 | 4.16E-14 | 4.755385 | up |
| LOC107801367 | 26 | 46 | 77 | 619 | 609 | 289 | 2.77E-05 | 3.585077 | up |
| LOC107803998 | 80 | 52 | 607 | 6416 | 7941 | 4076 | 1.19E-09 | 4.860855 | up |
| LOC107808944 | 50 | 48 | 63 | 114 | 171 | 119 | 0.002111 | 1.5336 | up |
| LOC107809178 | 12 | 31 | 193 | 855 | 1165 | 984 | 9.28E-24 | 3.871563 | up |
| LOC107815484 | 15 | 26 | 51 | 1131 | 1087 | 672 | 4.13E-13 | 5.20051 | up |
| LOC107816752 | 5324 | 5819 | 8166 | 9971 | 17807 | 13277 | 0.00576 | 1.289043 | up |
| LOC107817639 | 795 | 822 | 963 | 1711 | 2358 | 1431 | 0.004825 | 1.307452 | up |
| LOC107818832 | 148 | 70 | 145 | 6257 | 4151 | 934 | 0.002844 | 5.213553 | up |
| LOC107820286 | 0 | 11 | 58 | 374 | 486 | 122 | 0.001993 | 4.08102 | up |
| LOC107821207 | 46 | 84 | 428 | 1503 | 3080 | 2012 | 2.40E-06 | 3.767633 | up |
| LOC107822960 | 5 | 3 | 0 | 1224 | 630 | 204 | 0.000123 | 8.252878 | up |
| LOC107826470 | 3 | 9 | 82 | 422 | 419 | 300 | 1.61E-12 | 3.81783 | up |
| LOC107829152 | 121 | 139 | 135 | 263 | 394 | 233 | 0.006217 | 1.388337 | up |

FDR: false discovery rate; FC: fold change

**Supplementary Table 8.** DEGs (differentially expressed genes) involved in secondary cell wall biogenesis annotated by ClueGO

| Gene symbol | log2FC (JcFT / CK) | Regulated | Swiss_Prot_ or KEGG annotation | Type of IRX |
| --- | --- | --- | --- | --- |
| LOC107791241 | -1.252955078 | down | Protein trichome birefringence-like 16 |  |
| LOC107773919 | 1.956100865 | up | Protein REDUCED WALL ACETYLATION 3 |  |
| LOC107762480 | 3.978770563 | up | beta-1,4-xylosyltransferase IRX9 | IRX9 |
| LOC107764680 | 4.617732073 | up | beta-1,4-xylosyltransferase IRX9 | IRX9 |
| LOC107817758 | 5.476290583 | up | CESA4 | IRX5 |
| LOC107820514 | 4.78387138 | up | CESA4 | IRX5 |
| LOC107790572 | 4.268664404 | up | CESA4 | IRX5 |
| LOC107769133 | 5.421073638 | up | CESA7 | IRX3 |
| LOC107806628 | 5.344256523 | up | CESA7 | IRX3 |
| LOC107759843 | 6.154913333 | up | Protein IRX15-LIKE | IRX15L |
| LOC107790336 | 5.600006967 | up | Protein IRX15-LIKE | IRX15L |
| LOC107793555 | 4.85355889 | up | Protein IRX15-LIKE | IRX15L |
| LOC107803998 | 4.860854956 | up | Protein IRX15-LIKE | IRX15L |
| LOC107769571 | 2.027097236 | up | beta-1,4-xylosyltransferase IRX14H | IRX14H |
| LOC107796855 | 2.083023956 | up | beta-1,4-xylosyltransferase IRX14H | IRX14H |
| LOC107765203 | 3.22736915 | up | FLA11 | IRX13 |
| LOC107770483 | 3.835127591 | up | FLA11 | IRX13 |
| LOC107778308 | 3.594851727 | up | FLA11 | IRX13 |
| LOC107778315 | 5.45170023 | up | FLA11 | IRX13 |
| LOC107812975 | 3.342295478 | up | FLA11 | IRX13 |
| LOC107760089 | 2.176294476 | up | beta-1,4-xylosyltransferase IRX10L | IRX10L |
| LOC107793917 | 2.073854841 | up | beta-1,4-xylosyltransferase IRX10L | IRX10L |
| LOC107801367 | 3.5850772 | up | beta-1,4-xylosyltransferase IRX10L | IRX10L |
| LOC107817639 | 1.307451977 | up | beta-1,4-xylosyltransferase IRX10L | IRX10L |
| LOC107809178 | 3.87156268 | up | beta-1,4-xylosyltransferase IRX10 | IRX10 |
| LOC107828491 | 5.29186485 | up | CESA8 | IRX1 |
| LOC107830312 | 5.042746881 | up | CESA8 | IRX1 |
| LOC107762779 | Infinite | up | Fasciclin-like arabinogalactan protein 12 |  |
| LOC107770485 | 5.066906516 | up | Fasciclin-like arabinogalactan protein 12 |  |
| LOC107771244 | 3.792296273 | up | GXM1 |  |
| LOC107821207 | 3.767633462 | up | Glucuronoxylan 4-O-methyltransferase 1 | |
| LOC107772801 | 1.369414706 | up | Glucuronoxylan 4-O-methyltransferase 3 | |

FDR: false discovery rate; FC: fold change

**Supplementary Table 9.** Biological processes and primary protein types associated with differentially expressed genes (DEGs) that directly interacted with 14-3-3 proteins.

| Function/biological process | Numbers of DEGs | Type of protein |
| --- | --- | --- |
| 1. Signal transduction | 72 | Serine/threonine-protein kinase, receptor-like protein kinase |
| 2. Replication, recombination and repair | 31 | MCM^1^, DEAD-box helicase |
| 3. Cell cycle control, cell division | 11 | Calcium-binding protein KRP1, cyclin-A |
| 4. Translation, ribosomal structure and biogenesis | 18 | 40S ribosomal protein SA, 60S ribosomal protein L4 |
| 5. Posttranslational modification, protein turnover, chaperones | 20 | SKP1-like protein 4, heat shock protein |
| 6. Carbohydrate transport and metabolism | 6 | Fructose-2,6-bisphosphatase, xylose isomerase |
| 7. Energy production and conversion | 5 | Aldehyde dehydrogenase, sucrose non-fermenting protein |
| 8. Amino acid transport and metabolism | 7 | Ferredoxin-dependent glutamate synthase, glycine dehydrogenase |
| 9. Inorganic ion transport and metabolism | 11 | Sodium/hydrogen exchanger, phospholipid-transporting ATPase |
| 10. Intracellular trafficking, secretion, and vesicular transport | 25 | Ras-related protein, rac-like GTP-binding protein |
| 11. Cytoskeleton | 5 | Myosin-9, actin |
| 12. Cell wall/membrane/envelope biogenesis | 10 | Receptor kinase-like protein, serine/threonine-protein kinase |
| 13. Transcription | 47 | LRR receptor-like serine/threonine-protein kinase, heat stress transcription factor |
| 14. Molecular function regulator/binding | 20 | Zinc finger protein |
| 15. Plant hormone signal transduction | 4 | BES1/BZR1 homolog protein, protein brassinazole-resistant |
| 16. Function unknown | 2 |  |

^1^ MCM: minichromosome maintenance protein
